# Stress Reduction by Yoga versus Mindfulness Training in Adults Suffering from Distress: A Three-Armed Randomized Controlled Trial including Qualitative Interviews (RELAX Study)

**DOI:** 10.3390/jcm11195680

**Published:** 2022-09-26

**Authors:** Jan Moritz Fischer, Farid-Ihab Kandil, Christian S. Kessler, Lucas Nayeri, Laura Sophie Zager, Theresa Rocabado Hennhöfer, Nico Steckhan, Daniela A. Koppold-Liebscher, Holger C. Bringmann, Thomas Schäfer, Andreas Michalsen, Michael Jeitler

**Affiliations:** 1 Institute of Social Medicine, Epidemiology and Health Economics, Charité—Universitätsmedizin Berlin, Corporate Member of Freie Universität Berlin and Humboldt-Universität zu Berlin, 10117 Berlin, Germany; 2 Department of Internal and Integrative Medicine, Immanuel Hospital Berlin, 14109 Berlin, Germany; 3 MSB Medical School Berlin, 14197 Berlin, Germany

**Keywords:** yoga, iyengar yoga, mindfulness, meditation, stress reduction, mixed methods

## Abstract

Distress is a growing public health concern. In this three-armed randomized controlled trial, n = 102 adults with elevated stress levels and stress-related symptoms were randomly assigned to (1) “integrative” yoga classes which combined physical exercises, mindfulness training, and ethical/philosophical aspects of traditional yoga; to (2) Iyengar yoga classes which entailed primarily physical exercises; or to (3) mindfulness training without physical training. We hypothesized the synergistic effects of physical yoga exercises, mindfulness, and ethical/philosophical aspects. The primary outcome was the group difference on Cohen’s Perceived Stress Scale (PSS) after 12 weeks. Secondary outcomes included burnout, quality of life, physical complaints, depression, anxiety, mindfulness, interoceptive awareness, self-regulation, spirituality, mysticism, and posttraumatic stress. All outcomes were evaluated at baseline (V0), after 12 weeks (V1), and after 24 weeks (V2). A subset of participants took part in qualitative interviews. A lasting and clinically relevant stress reduction was observed within all groups (PSS ΔV0–V1_Integrative Yoga_ = −6.69 ± 6.19; ΔV0–V1_Iyengar Yoga_ = −6.00 ± 7.37; ΔV0–V1_Mindfulness_ = −9.74 ± 7.80; all *p* < 0.00). Effect sizes were also statistically large at the end of the follow-up period (Cohen’s d _Integrative Yoga_ = 1.41; d _Iyengar Yoga_ = 1.37; d _Mindfulness_ = 1.23). There were no significant group differences or evidence of relevant synergistic effects from combining mindfulness and physical yoga exercises. All three interventions were found to be equally effective methods of stress reduction. Their use in practice should be based on availability and patient preference.

## 1. Introduction

Stress has been defined as a real or anticipated disruption of the homeodynamic balance or an anticipated threat to well-being [1]. It can be caused by a wide range of intrinsic or extrinsic stimuli or stressors. Stressors can be real or perceived; thus, stress can be physical, but also purely psychological [2].

Two of the major physiological systems of stress response include the hypothalamic–pituitary–adrenocortical (HPA) axis and the autonomic nervous system (ANS). In contrast to repeated, ephemeral, and motivating stress, inadequate, aversive, excessive, or prolonged stress may exceed the regulatory capacity and adjustive resources of the organism. Chronic stress can, thus, cause sensitization, as well as habituation, of the HPA axis and ANS responses [1]. In consequence, maladaptive responses are produced, as well as chronically altered homeodynamic capacities, associated with negatively affected mental health, physical health, and life expectancy [2,3,4].

Consequently, increased stress is a significant risk factor for the most common chronic diseases [5,6]. Acute and chronic stress is implicated in the development of hypertension, coronary heart disease, general cardiovascular mortality, infectious diseases, chronic inflammation, chronic pain, fatigue syndrome, obesity, diabetes type II, metabolic syndrome, osteopenia/osteoporosis, headache, and cancer, among others [7,8,9,10,11,12,13,14,15]. Furthermore, it is linked to a broad range of psychiatric disorders, including anxiety, depression, eating disorders, post-traumatic stress disorder, and sleep disorder [12,13,14,15,16,17,18]. Stress, therefore, results in estimated annual costs of more than 1% of the gross domestic products of Western countries [19,20,21].

It has been shown that relaxation techniques can interrupt a chronic stress load, and, thus, help avert its negative consequences [22,23]. Moreover, they can enable changes in cognition, thus reducing perceived stress [22,23,24,25,26]. Various techniques have been shown to be effective and are routinely applied in prevention, therapy, and rehabilitation [27,28]. Particularly prevalent is the use of mindfulness-based methods such as mindfulness meditation and yoga [29,30,31].

Mindfulness describes focusing on the present moment. Body perceptions, thoughts, and emotions are met with a non-judgmental and accepting attitude [32,33]. Its psychological mechanisms of action are well researched. By training the ability to focus on the present, it helps to disrupt dysfunctional mental processes [22,25,26,34,35]. On a neuropsychological level, areas of the brain involved in emotion regulation are activated and trained [36]. Ultimately, improved attention regulation can lead to the ability to control thoughts, feelings, and behavior better [24,25,37]. The dysfunctional experience and management of stress, which is associated with poor emotion regulation, negative cognitions, and impulsive behavior, can be changed [24,32,38,39,40,41,42,43]. Subjectively perceived stress then decreases, allowing for increases in physical and mental health and in quality of life [42,44,45,46,47,48].

Yoga originated in South Asia more than 2000 years ago. It traditionally integrates religious, spiritual, physiological, and psychological methods into a “Whole Medical System” [49]. There are different styles of yoga that emphasize certain sub-aspects. This diversity makes it difficult to standardize its mechanisms of action, and to study yoga in general [50]. Most research on yoga as a medical tool focuses on less complex “body-oriented” forms of yoga such as Iyengar yoga. More complex forms are poorly studied in terms of medical outcomes, including stress reduction. The results of the few available studies examining integrated yoga show positive effects regarding stress experience, anxiety, depression, quality of life, and physical illness [51,52]. Practitioners may benefit from the synergistic effects of different aspects of yoga [53,54]. In this study, an attempt is made to reflect a complex kind of yoga in the sense of a Whole Medical System under the name “integrative yoga”, including physical yoga exercises, mindfulness, and ethical/philosophical aspects [55].

Iyengar yoga is a widely recognized and well researched style of yoga. In its initial stages, Iyengar yoga focuses largely on the physical practice of yoga postures, and can, therefore, be described as “body-oriented”. It was established from 1937 on by its founder, B.K.S Iyengar. He systematically studied the benefits of various yoga exercises, and introduced assistive devices so that people with different physical conditions could perform the exercises [56]. His scientific approach contributed significantly to the researchability of yoga [57]. In our own studies with women suffering from stress, pronounced beneficial effects on quality of life, anxiety, stress, and depression were found after 12 weeks of intensified Iyengar yoga practice [57,58]. Another study showed evidence of anxiolytic and antidepressant effects of Iyengar yoga [59]. In the context of this study, Iyengar yoga exercises were taught without meditation and philosophical content to reflect only the physical aspect of yoga.

Mindfulness training, integrative yoga, and Iyengar yoga differ significantly in terms of content and physical engagement. This leads, in turn, to differences in clinical applicability and, possibly, efficacy. In integrative yoga, meditation, physical movement, and ethical/philosophical aspects are combined with the principles of mindfulness, possibly enhancing efficacy [60,61]. Mindfulness training, in comparison, has very low physical requirements for the practitioner [38]. Although the psychological mechanisms of action of mindfulness are well researched, it is unclear whether mindfulness-based relaxation techniques develop their effect solely through an improved capacity for mindfulness [39].

Against this background, the present study investigates the extent to which the above-mentioned relaxation methods differ from one another in their effects. Of particular interest is whether there are indications of a special effectiveness when physical exercises are combined with mental mindfulness exercises.

**Hypothesis 1.**  *Stress reduction by integrative yoga > stress reduction by Iyengar yoga > stress reduction by mindfulness training.*

We also examine whether stress reduction, along with any other effects, differentially affects quality of life. In doing so, we assume an increased effectiveness depending on the number of potential effect factors of each method.

**Hypothesis 2.**  *Quality of life by integrative yoga > quality of life by Iyengar yoga > quality of life by mindfulness training.*

To adequately map the broader, multifaceted effects of integrative yoga, Iyengar yoga, and mindfulness training, we also conducted qualitative interviews. These were intended to provide a more accurate understanding of how study participants experienced each intervention. Moreover, they were to provide information on how the interventions may have changed the participants’ perception of stress in daily life. The findings are intended to further elucidate the mechanisms of stress reduction, and might allow for a more targeted use of yoga and/or mindfulness training in clinical psychological practice.

## 2. Materials and Methods

### 2.1. Study Design

**Figure 1:**
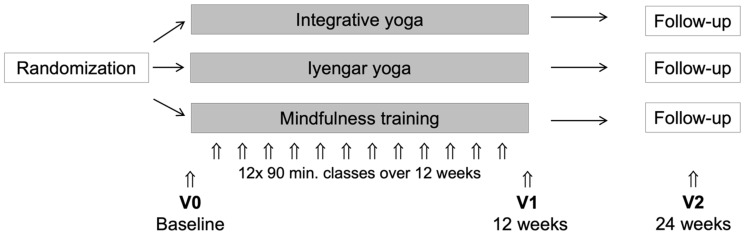
Study structure.

We conducted a single-center, three-arm, randomized, controlled clinical trial combined with qualitative interviews. N = 102 participants with elevated perceived stress levels and physical stress-related symptoms (e.g., muscle tension) were randomly assigned to (1) integrative yoga classes which combined physical exercises, mindfulness training, and ethical/philosophical aspects of traditional yoga; to (2) Iyengar yoga classes which focused on physical exercises; or to (3) mindfulness training without any physical exercises. The allocation ratio was 1:1:1. Each group received the same amount of instruction and daily exercise over a period of 6 months (12-week intervention period, 12 weekly classes of 90 min each, followed by 12-week follow-up period; Figure 1). A home exercise practice was recommended for participants in all groups (30 min daily; also during the follow-up period). The primary outcome was the group difference on the Perceived Stress Scale by Cohen (PSS) after 12 weeks. Secondary outcomes included burnout, quality of life, physical complaints, depression, anxiety, mindfulness, interoceptive awareness, self-regulation, spirituality, mysticism, and posttraumatic stress. All outcomes were assessed at baseline, after 12 weeks, and after 24 weeks (follow-up) by validated questionnaires in the German language. In addition, adherence was monitored weekly via an online questionnaire for 12 weeks (practice time in minutes per week).

A randomly chosen subset of participants from each group participated in qualitative interviews. Semi-structured individual interviews were conducted during the follow-up period. A mixed-method approach was chosen to enable a deeper understanding of patients’ experiences with each relaxation technique, and to generate future hypotheses about their mode of action. Initially, progressive muscle relaxation was supposed to be the non-physical control instead of mindfulness training. Popularity for this intervention was very low among potential study participants during early recruitment. Therefore, mindfulness training was introduced as a replacement before interventions started. The study registration and ethics approval were amended accordingly.

### 2.2. Recruitment

Patients were recruited from July 2019 until January 2021 in Berlin, Germany. The study was advertised for in Berlin public transport, on social media, on the premises of the Charité University hospital via flyers, and on the website of the Charité Outpatient Clinic for Integrative Medicine. Volunteers were screened on the telephone, and later checked for eligibility by a study physician. Baseline assessment directly followed if patients were eligible (Table 1).

### 2.3. Randomization

Patients were enrolled and randomly assigned to one intervention after baseline assessment by a study physician. The randomization list had been generated by an independent researcher from another project, using blockrand library (v 1.4) with varying block lengths in R (v 3.5). The sequence was not known to the study physicians. Intervention groups were filled until each group had at least four participants. Then, a 12-week program started for one cohort. Recruitment was ongoing. In total, there were four cohorts of three groups each.

### 2.4. Setting

Yoga/meditation classes were taught exclusively for the study participants by certified instructors in commercial studios for each integrative yoga, Iyengar yoga, and mindfulness training. The instructors for integrative yoga were required to have completed a 4-year program by the Professional Association of Yoga Teachers in Germany (BDY/EYU). The instructors for Iyengar yoga were required to have a certification of Iyengar Yoga Germany (IYD), a program which involves 1000 h of training. The instructors for mindfulness training had to be qualified psychologists with experience in mindfulness meditation. Applicable studios in central Berlin were asked to support the trial. Three studios were chosen based on their willingness to participate and accessibility. All instructors taught exclusive classes of 4–12 study participants once per week for 12 weeks. Every session lasted 90 min. The premises of all studios were of comparable dimensions, lit, unfurnished, painted in neutral colors, quiet, and warm. The content of each lesson was determined in advance in collaboration with the study physicians and logged. The teaching instructors remained the same during every 12-week program. No evaluation or examination was carried out by the instructors. Only the 1st cohort with 15 participants took place on site; all other 3 cohorts were offered online due to the COVID-19 pandemic.

All examinations were carried out by the study physicians and took place in the Charité Outpatient Clinic for Integrative Medicine. In the first cohort, study participants visited the study center for examinations at baseline, and at the end of the 12-week intervention. However, due to the COVID-19 pandemic, on-site study visits were not possible, so these were conducted online in the following cohorts.

### 2.5. Interventions

#### 2.5.1. Integrative Yoga

Participants in group 1 received an established integrative yoga program in the sense of a “Whole Medical System”. “Whole Medical System” refers to an independent, comprehensive, and self-contained philosophy about health, including an owned (medical) practice [62]. Classes contained meditation and relaxation techniques, as well as the exercise of yoga postures (asanas), breathing techniques (pranayama), and ethical/philosophical aspects of yoga (Supplementary Materials: Standard Operating Procedures-Integrative Yoga Practice). The methods and content could also be tailored specifically to the individuals’ wishes and needs to further reflect the idea of integrative yoga [55]. Therefore, the operationalization was supposed to reflect the inherent adaptability of yoga practice [51,52,63]. The integration and combination of the individual contents of yoga was to allow for a concentration of positive effects.

#### 2.5.2. Iyengar Yoga

The participants of group 2 received an established yoga program based on the internationally renowned yoga school of B.K.S. Iyengar [56]. Classes focused on the physical aspects of yoga and did not entail meditation or the ethical and philosophical aspects of yoga. They focused on executions of yoga postures (asanas) and a final relaxation (Shavasana; Supplementary Materials: Standard Operating Procedures—Iyengar Yoga Practice). In accordance with the teachings of B.K.S. Iyengar, assistive devices could be used when practicing yoga postures. A similar program had produced increases in quality of life and reductions in anxiety, stress, and depression in a prior study [57].

#### 2.5.3. Mindfulness Training

The participants in group 3 received mindfulness training on healthy stress management by a trained psychologist. The program was based on the principles of Mindfulness-Based Stress Reduction (MBSR) by Kabat-Zinn [64], but was expanded to 12 weeks to match the other two intervention groups. In addition, the “mindful stretching” portion included in the original program by Kabat-Zinn was omitted. The classes included mindfulness-based meditation and practical strategies for stress management (Supplementary Materials: Standard Operating Procedures—Mindfulness Training). The main goal was to improve the perception of inner processes and needs by practicing a conscious, moment-oriented, and open attitude. This was supposed to enable an early and adequate response to stress, and, thus, reduce perceived stress in general [65].

### 2.6. Primary Outcome

The main endpoint, the subjective stress experience, was examined using the Perceived Stress Scale that was originally developed by Cohen, Kamarck, and Mermelstein in 1983 [66]. It is based on Lazarus’ psychological stress model [67]. The scales are intended to measure the extent to which life situations have been experienced as stressful in the past month, especially the extent to which a person feels that everyday coping is characterized by unpredictable, uncontrollable, and over-stressful experiences. In addition, it assesses whether the individual stress limit has been exceeded. The assessment of the subjective stress experience is made on a scale of never (1), almost never (2), sometimes (3), quite often (4), and very often (5). The original 14 items are applicable to the general population due to their universality [68]. The PSS has high internal consistency and test–retest reliability [66]. Due to the superiority of the reliability of the 10-item short version (Cronbach α = 0.78 vs. Cronbach α = 0.75), the applicability of this scale in clinical settings is indicated [68]. The German translation used has been validated [68,69].

### 2.7. Secondary Outcomes

#### 2.7.1. Maslach Burnout Inventory (MBI)

Burnout syndrome can be a consequence of prolonged stress exposure [70]. The Maslach Burnout Inventory (MBI) is the most used tool in research on burnouts, and assesses three components with 22 items of burnout syndrome: cynicism, exhaustion, and reduced professional efficacy [71,72]. A validated German version (MBI-D) was being used [73,74].

#### 2.7.2. Short Form 36 Health Survey (SF-36)

The Short Form 36 Health Survey (SF-36) is an internationally renowned tool to assess quality of life in medical, psychological, and economic research [75]. It provides a comprehensive assessment of a person’s health status in 8 dimensions, ranging from physical functioning to emotional and social functioning. These can be summarized to a mental and to a physical component summary score [76].

#### 2.7.3. Beschwerden-Liste/Zerssen Symptom List (B-LR and B-LR’)

The Zerssen Symptom List was developed as a self-assessment tool for general and somatic symptoms [77]. Two parallel forms exist, which are closely correlated with each other (r ≈ 0.9). They allow an assessment of the subjectively perceived physical well-being.

#### 2.7.4. Hospital Anxiety and Depression Scale (HADS)

The Hospital Anxiety and Depression Scale (HADS) is a clinically meaningful 14-item psychological instrument used for screening states of depression and anxiety in the setting of a hospital medical outpatient clinic [78,79]. The HADS allows tracking the course of diseases and the response to psychotherapeutic and psychopharmacological intervention over time [80]. It is recognized as an economical, reliable, and sufficiently valid self-assessment method [81,82]. A validated German Version (HADS-D) has been used [81].

#### 2.7.5. Freiburg Mindfulness Inventory (FMI)

The Freiburg Mindfulness Inventory was developed to reflect the concept of mindfulness, including its Buddhist tradition [83]. The items consist of statements such as “I accept unpleasant experiences” and are rated on a 4-point frequency scale from “almost never” to “almost always”. The 14-item version has been validated for both individuals who meditate regularly and for normal samples. The original German version has a high internal consistency and validity [84].

#### 2.7.6. Multidimensional Assessment of Interoceptive Awareness (MAIA)

The Multidimensional Assessment of Interoceptive Awareness reflects 8 dimensions of mind–body interaction or body awareness [85]. It is used in research to evaluate the effects of diseases such as major depression or fibromyalgia on mind–body interaction [86,87].

#### 2.7.7. Self-Regulation Inventory (SRI)

The Self-Regulation Inventory consists of 16 questions concerning the assessment of self-regulation according to Grossarth-Maticek [88]. The items have a scale from 1 (very weak) to 6 (very strong), and are calculated into a sum value. The questionnaire was applied and tested regarding validity and reliability [89]. It was found to have excellent internal consistency (Cronbach α = 0.948) and sufficient test–retest reliability (r = 0.796) [90].

#### 2.7.8. Aspects of Spirituality (ASP)

The Aspects of Spirituality questionnaire is an open 40-item multidimensional construct to measure the distinct expression of spirituality with both religious and secular forms [91,92]. The questionnaire is suited for skeptical, areligious, and religious individuals [93]. It was used to monitor possible changes in spirituality, especially in the integrative yoga group, whose participants were also exposed to spiritual content (these were communicated in an ideologically neutral way).

#### 2.7.9. Hood’s Mysticism Scale (HMS)

Hood’s Mysticism Scale is a questionnaire that allows the quantification of spirituality and the perceived attainment of insight [94]. Its three-factor structure has been validated for followers of different faiths [95]. The 8-item short form was used in this study [96].

#### 2.7.10. Posttraumatic Stress Disorder Checklist for DSM-5 (PCL-5)

The Posttraumatic Stress Disorder Checklist was used to monitor possible inherent posttraumatic stress. It comprises 17 items which resemble the PTSD symptom criteria [97]. It is the most widely used measure of posttraumatic stress disorder and has high internal consistency (α = 0.94), test–retest reliability (r = 0.82), and discriminant (rs = 0.31 to 0.60) validity [98].

### 2.8. Sample Size

For the 12-week comparison of the groups, an effect size of f = 0.15 (corresponding to a Cohen’s d = 0.3) was assumed in favor of integrative yoga. An optimal sample size per group of n = 30 was calculated for a repeated measures ANOVA, of which only the interaction between time × groups would be evaluated, further with 80% statistical power, and a correlation between measures of 0.6 (estimated from previous studies) and an alpha of 0.05. The total optimal sample size of n = 90 was increased by 20% to compensate for dropouts. The recruitment target was, therefore, n = 108.

### 2.9. Statistical Methods

The data collected were analyzed using SPSS 25. Outliers were supposed to be identified by visually checking raw boxplots. No data were excluded as a result. Missing values were imputed (MICE algorithm). Descriptive statistics were applied to present the sample characteristics. To test the hypotheses that integrative yoga is superior to Iyengar yoga and mindfulness training in terms of stress reduction and health-related quality of life, individual differences (gains) were first calculated, followed by a single-factor analysis of variance. As the number of 34 patients in each group was larger than 30, the distribution of the data was assumed to be asymptotically normal. Results are given as F, p, and eta^2^ effect sizes (with small, medium, and large effects assessed for eta^2^ ≥ 0.01, 0.06, and 0.14, respectively). Multiple *t*-tests were employed in an exploratory manner to present changes (gains) within each of the three groups separately. Results here are given as T, p, and effect-size Cohen’s d (with small, medium, and large effects assessed for Cohen’s d ≥ 0.20, 0.50, and 0.80, respectively (Cohen and Sawilowsk [99,100]). All statistical analyses were performed as intention-to-treat analyses. 

### 2.10. Qualitative Approach

The study questions of how the interventions were experienced, and in what ways they potentially affected the subjects’ experience of stress in their daily lives, required a qualitative design [101]. We chose semi-structured individual interviews in conjunction with a content analysis according to Mayring [102]. Interviewees should have been present in at least 6 out of 12 classes to ensure a minimum level of exposure. From all study participants who met this requirement and were interested, participants were randomly selected for the qualitative interviews. All study participants received an invitation to volunteer in the interviews. Participation was incentivized with a book voucher worth 10€. In the absence of theoretical saturation, more interviews could have followed.

The two researchers involved in the qualitative part of the study (JMF and TRH) each contemplated their own experience and beliefs regarding stress, mindfulness, and yoga. None of them had prior experiences with yoga or mindfulness, but they reported formative personal experiences with stressful experiences in college and academia. Both reported considering stress exposure to be a relevant problem and acknowledged the possibility of being personally influenced in this regard.

The interview guideline was created according to the study questions. It contained a brief explanation of the interview process for the participants to create an open and judgment-free discussion atmosphere. This was followed by three open questions on the subjective experience of the respective intervention, possible changes in everyday life, and an outlook for the future. Each question was accompanied by key points on possibly relevant topics that could be asked additionally (Supplementary Materials: Interview Guide).

Due to COVID-regulations, the interviews were held online. There were no time limits, and 30 min was estimated. The raw data were transcribed, and a semantic transcription of the content was carried out. The interviews were transcribed uniformly, word for word. Filler words such as “hmm” were removed, and dialects were translated into plain German.

Data evaluation and category building were performed according to Mayring’s content analysis [102]. In this work, the focus was only on verbal communication. Body-language-specific elements were not considered. The categories were formed inductively [103]. MAXQDA^®^ software (version 2018.2, Berlin, Germany) was used for the analysis.

In the initial systematic coding of the material, the texts were carefully read sentence by sentence, and sections of meaning were recorded, their meaning identified, and a code was formed from this. This was carried out independently by JMF and TRH to achieve a degree of intersubjectivity. Moreover, at this point, both researchers independently concluded that a theoretical saturation had been reached. No further interviews were conducted.

The initial rough coding identified and summarized relevant passages from the interviews. Simple sentences, sections of meaning, or short dialogues counted as codes. Single statements could be coded several times, and a section could be assigned to an already existing code. The codes were structured, combined, and assigned to different topics. In this way, potential topics and subtopics were compiled, and an overview of possible relationships between these topics was created. A review, restructuring, and revision of the codes took place until a meaningful structure emerged and an overview of the themes and their relationships to each other could be depicted. The resulting category system allowed to identify relevant themes and their relation to each other. In the last step of the analysis, an overview of all relevant data (category system and content) was examined considering the research questions. For this purpose, individual statements were paraphrased concisely and limited to the content in the summary analysis. In this paraphrasing, text components that were unimportant for the content were omitted, and those that occur frequently were summarized [102].

## 3. Results

### 3.1. Quantitative Results

**Figure 2:**
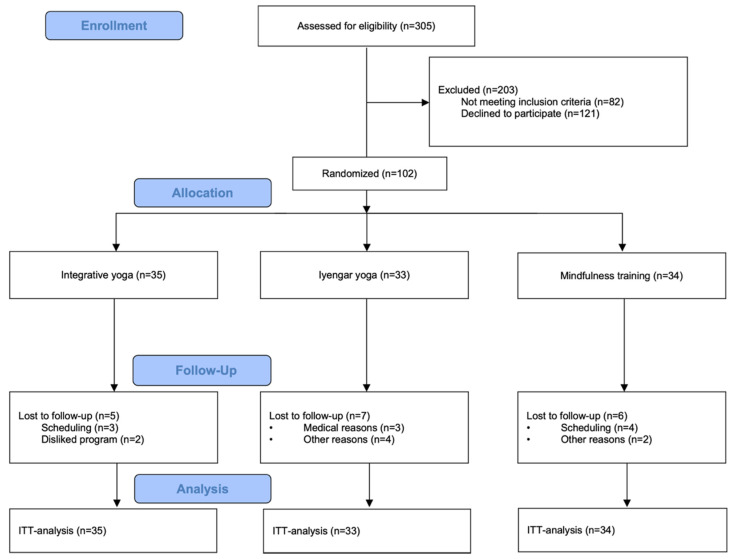
Participant flow.

The trial ended after the planned number of participants reached the end of the follow-up period. We analyzed the data of n = 102 study participants in the intention-to-treat analysis. Three-hundred and five volunteers were initially screened for eligibility, and two-hundred and three were excluded prior to randomization (Figure 2). One-hundred and two were randomized and did receive an intervention. Out of 35 participants assigned to the integrative yoga group, 30 completed the study. Three dropped out because they felt regular attendance only added to tightening their daily schedules. Two dropped out because they felt the program was not effective. Thirty-three participants were assigned to the Iyengar yoga group, twenty-six of which completed the study. A total of seven were lost to follow-up, three because regular attendance added to their stress, three because they felt the Iyengar yoga program was not working for them, and one person reported she did not get along with her instructor. Thirty-four participants were assigned to the mindfulness training group, and twenty-eight of them completed the study. Six were lost to follow-up, four because they struggled with regular attendance, one because they felt mindfulness training was not effective, and one because they were dissatisfied with the group they were randomly assigned to. No participants changed intervention groups.

When the COVID-19 pandemic hit Germany in early 2020, all classes were seamlessly converted to an online format (live-streaming of the classes). Single patients had technical difficulties at first, which could be resolved with the help of the instructors and the study-personnel. The online format was fully implemented for every participant after a transitional period of two weeks. The overall mean attendance for the 12 weekly training sessions in each group was: integrative yoga: 9.0 ± 2.3 sessions; Iyengar yoga: 7.2 ± 3.1 sessions; mindfulness: 8.0 ± 4.2 sessions.

A total of 18 participants was lost to follow-up across all groups. Missing data were checked for systematic bias. Little’s test was non-significant with a Chi^2^ of 253.3 (at df = 350 degrees of freedom); *p* = 0.999. Therefore, it was assumed that data were missing completely at random.

Baseline characteristics in all groups were similar, as a sign of successful randomization (Table 2). As expected, the primary outcome of perceived stress (PSS) was more than two standard deviations higher before the intervention in all groups (PSS scores: integrative yoga: 33.91 ± 4.80; Iyengar yoga: 33.39 ± 5.91; mindfulness: 34.44 ± 5.84) compared with the general population (PSS in the general population: age 20–39: 12.74 ± 6.67; age 40–59: 12.82 ± 6.42) [68].

**Figure 3:**
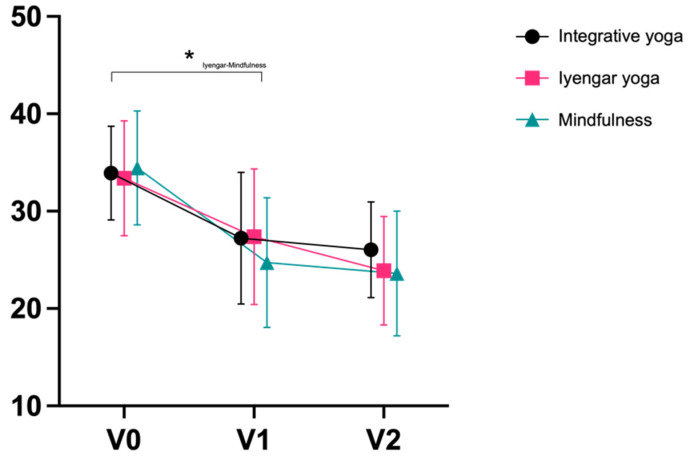
Perceived Stress Scale (Mean ± SD). * = *p* <0.05.

The primary outcome, PSS, decreased similarly post-intervention in all groups. Effect sizes ranged from large (Cohen’s d = 1.08 integrative; d = 0.81 Iyengar) to very large (d = 1.25 mindfulness). Effect sizes were very large in all groups after the end of the follow-up period, and ranged from d = 1.23 mindfulness to d = 1.41 integrative (Table 3; Figure 3). There were no significant differences between groups except for a superiority of mindfulness training over Iyengar yoga from V0 to V1 (*p* = 0.048; η^2^ = 0.057). Differences from V0 to V2 were not statistically significant between all groups. In all groups, a slightly greater reduction in stress (PSS) was observed with longer self-reported exercise time in n = 76 participants (n = 28 integrative; n = 23 Iyengar; n = 23 mindfulness); however, the significance level was not reached (r = 0.167, *p* = 0.156; Supplementary Materials: Plot Practice Time).

**Figure 4:**
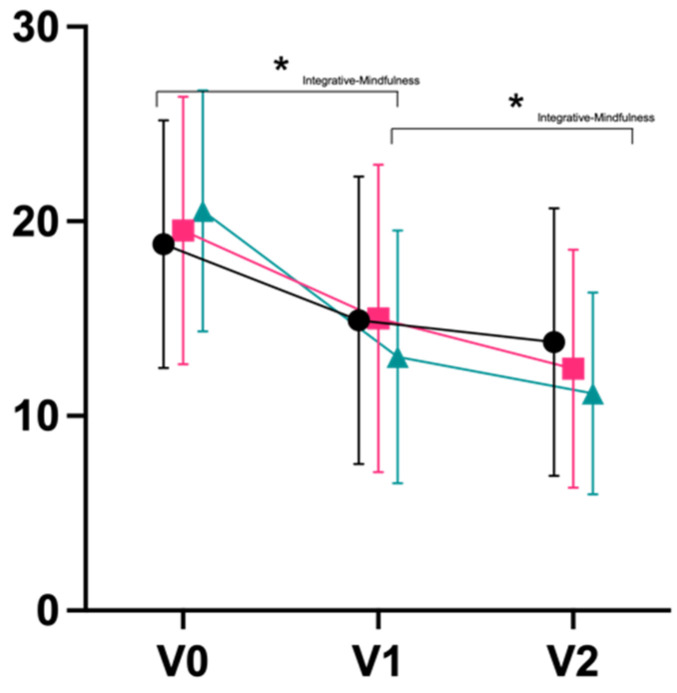
Exhaustion MBI (Mean ± SD). * = *p* < 0.05.

**Figure 5:**
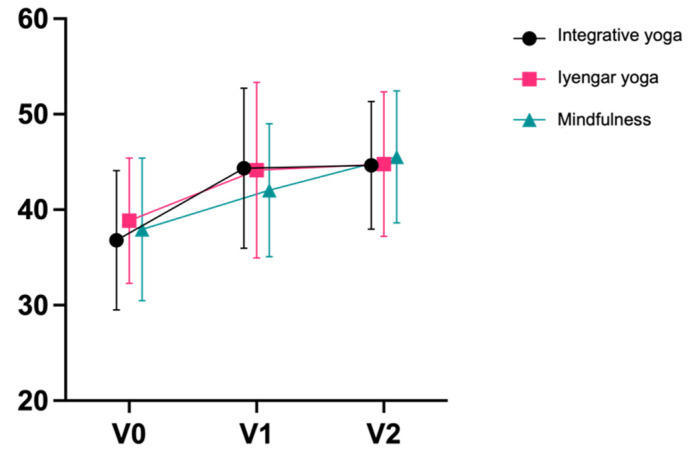
Mental Component—Quality of Life SF-36 (Mean ± SD).

**Figure 6:**
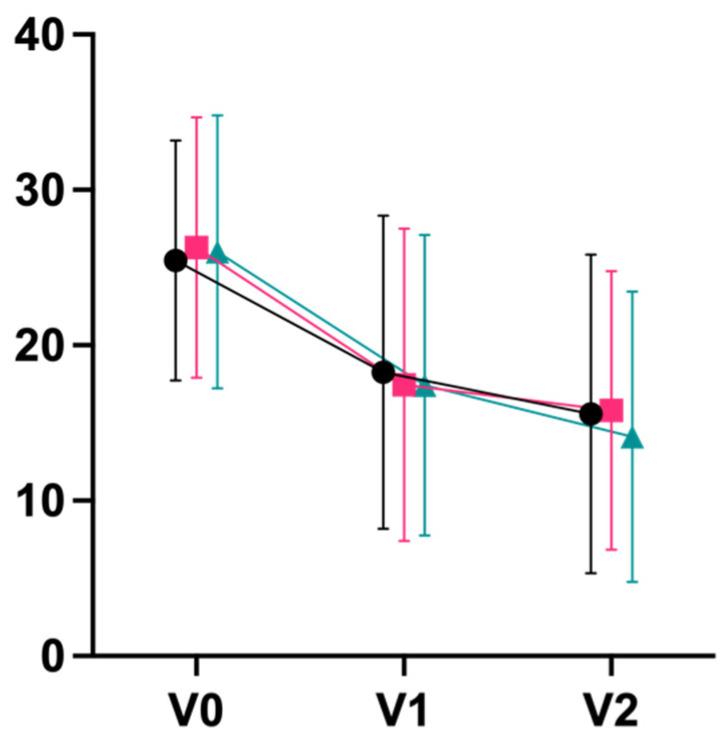
Physical Wellbeing B-LR (Mean ± SD).

**Figure 7:**
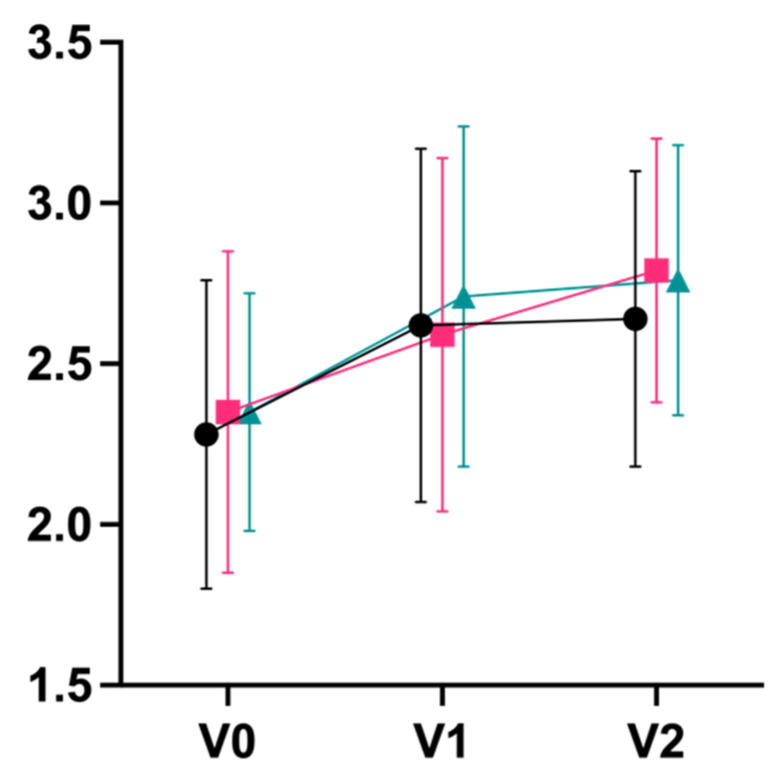
Mindfulness FMI (Mean ± SD).

**Figure 8:**
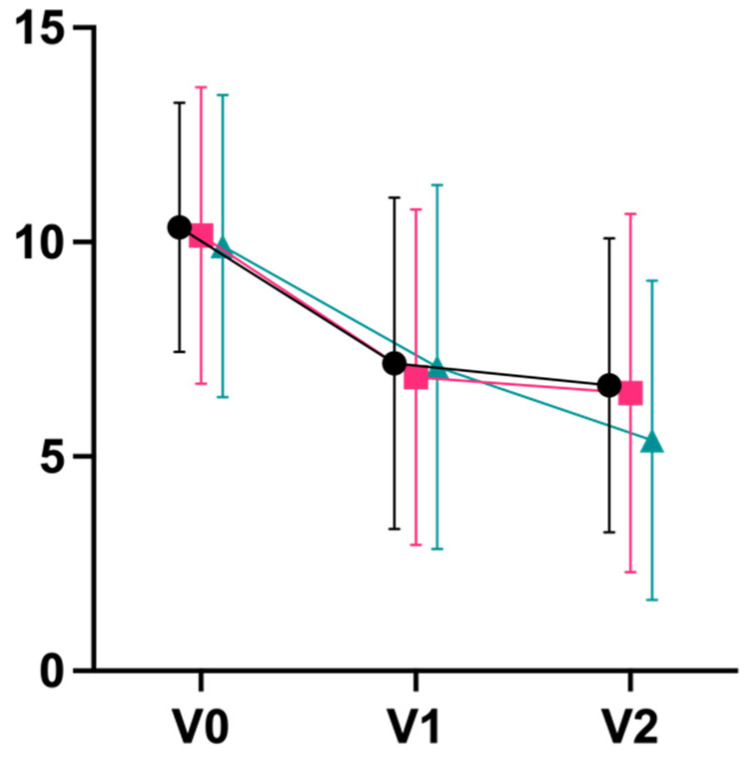
Anxiety HADS (Mean ± SD).

**Figure 9:**
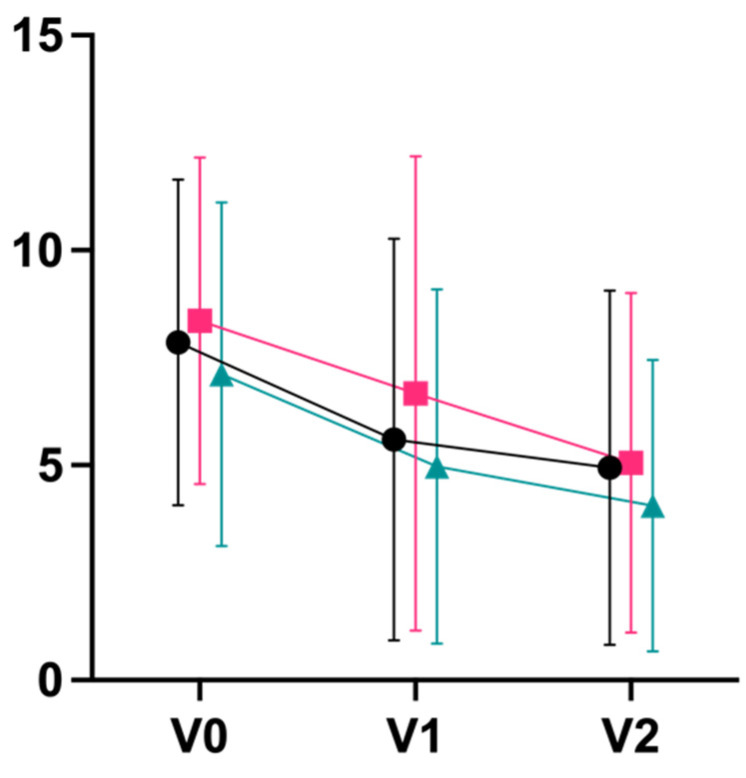
Depression HADS (Mean ± SD).

For secondary outcomes, we observed large effects in terms of decreases on the exhaustion subscale of the Maslach Burnout Inventory (d_V0–V2_ 0.80 integrative; 0.94 Iyengar; 1.13 mindfulness), with a statistically significant superiority of mindfulness meditation over integrative yoga post-intervention (p_V0–V1_ = 0.044) and after follow-up (p_V0–V2_ = 0.017; Figure 4). Large effect sizes were also observed for an improvement on the mental component summary score of the quality-of-life questionnaire, SF-36 (d_V0–V2_ 1.13 integrative; 0.74 Iyengar; 0.81 mindfulness; Figure 5). Physical well-being (B-LR) improved with a large-to-very-large effect size in all groups (d_V0–V2_ 1.34 integrative; 1.28 Iyengar; 1.13 mindfulness; Figure 6). Mindfulness (FMI) increased with borderline-large-to-large effect sizes in all groups (d_V0–V2_ 0.73 integrative; 0.81 Iyengar; 0.73 mindfulness; Figure 7). Both subscales of the Hospital Anxiety and Depression Scale (HADS) improved in all groups with moderate-to-large effect sizes (subscale anxiety: d_V0–V2_ 0.89 integrative, 0.79 Iyengar, 0.92 mindfulness, Figure 8; subscale depression: d_V0–V2_ 0.71 integrative, 1.05 Iyengar, 0.68 mindfulness, Figure 9). 

Moderate-to-large effect sizes were also observed on the self-regulation subscale of the interoceptive body awareness questionnaire (MAIA) (d_v0–v1_ 0.89 integrative; 0.52 Iyengar; 0.88 mindfulness) and on the trusting subscale (d_v0–v2_ 0.61 integrative; 0.62 Iyengar; 1.06 mindfulness). Small-to-moderate effect sizes were found for the other subscales (noticing, not distracting, not worrying, attention regulation, emotional awareness, and body listening). Small-to-very-small effect sizes were observed for both the spirituality questionnaire (ASP) and the mysticism questionnaire (HMS). Posttraumatic stress (PCL-5) improved in all groups, with medium-to-borderline-medium effect sizes by the end of the follow-up period in all groups (d_v0–v1_ 0.49 integrative; 0.56 Iyengar; 0.73 mindfulness). Among these, there were no relevant statistically significant differences between groups, except for a weak superiority (small vs. very small effect size) of both integrative yoga and mindfulness meditation over Iyengar yoga on the cynicism subscale of the Maslach Burnout Inventory (MBI) post-intervention (p _integrative–Iyengar_ = 0.017; p _mindfulness–Iyengar_ = 0.036). Moreover, there was a superiority of mindfulness over Iyengar yoga on the search for wisdom subscale (medium vs. very small effect size) and on the religious orientation subscale (small vs. very small effect size) of the Aspects of Spirituality questionnaire (ASP) post-intervention. These three group differences were no longer significant at the end of the follow-up period (Supplementary Materials: Results—Randomized Controlled Trial).

### 3.2. Qualitative Results

Out of the study participants who had attended at least six out of twelve classes and volunteered to participate in the qualitative interviews, six were chosen at random (Table 4). The interviews were conducted in November and December 2020.

Using Mayring’s content analysis, three main categories with thirteen subcategories were formed (Table 5).

In the following, the results of the content analysis are summarized and presented, including some selected examples (Supplementary Materials: Results—Qualitative Interviews for a comprehensive presentation of qualitative results). All interventions were reported to allow time in daily life to be set aside for one’s own well-being.

*“My daily life is, it went on as it was before, or still is now. But I include an hour for myself.”*

(Participant 5—Iyengar yoga)

Participants from all groups reported that it also encouraged them to be proactive about their own well-being.

*“But a little bit, I have achieved that I allow myself more. […] I have a Jacuzzi in the bathroom, I’ve never used it, for 15 years, […] because I didn’t allow myself that. […] because that was a pampering for me and I’m not allowed to do that. I’ve overcome those little things now and that’s what I do now.”*

(Participant 6—Mindfulness training)

Participants from the mindfulness group and integrative yoga group reported that they had started to consciously practice mindfulness in their everyday life because they found it helpful for their well-being.

*“Yes, so this mindfulness of what’s happening to me and where I am right now and I’m trying to control that better. […] And now I always try to be aware of how each step is happening, what I’m doing and try not to get distracted. That’s not always successful, but that’s what I try to do.”*

(Participant 6—Mindfulness training)

The reported psychological changes were mainly related to stress management. The participants felt that they were generally better able to distance themselves from demands and were more relaxed; the degree varied.

*“Yes, so a little bit. Not as much as I would have liked, and as I’ve heard with the other women.”*

(Participant 6—Mindfulness training)

Both participants from the mindfulness group also reported very deep emotional confrontations with themselves. In one case, meditating and practicing relaxation exercises supposedly helped with both the grief processing of the death of a family member, as well as to question and alter learned structures of upbringing. In the other, it enabled a change in the participant’s relationship with himself, which was perceived as emotionally significant.

*“There were also moments when I really noticed a fundamental change. And where I really became aware that I had done too much to myself before. And one of the greatest moments was when, after a meditation, I stood there on my mat and started to cry and asked my body out loud for forgiveness for what I had done to it for 20 years in the job in which I now work. Nobody had told me to do that, that came from within, and I was very aware of that at that moment, that that came from me. And there were a few things like that then, during the course and even later.”*

(Participant 2—Mindfulness training)

Reported somatic health changes varied widely. Participants from the mindfulness group and from the Iyengar yoga group expressed disappointment that stress-associated conditions (tremor, back pain) did not change during the study. At the same time, participants from the integrative yoga group and from the mindfulness group reported a marked improvement of preexisting psoriasis, migraine, and back aches. One participant from the mindfulness group had lost 5 kg of excessive body weight. Eating had been a stress-coping mechanism for him. No perceived changes in flexibility or general physical fitness were reported spontaneously, with the exception of one participant from the integrative yoga group. She reported less neck pain due to increased neck mobility. Interestingly, participants from both yoga interventions expressed that yoga practice is a process that generally takes time to facilitate positive change.

All classed were described as positive overall, even by participants who expressed they would not take part again. The criticism focused on failed expectations and the monotony of the respective programs. One Iyengar participant wished for more exercises and poses to choose from. One participant from the integrative yoga group found the same sequence of poses throughout the class boring. One participant from mindfulness training and one from the Iyengar group found the exercises not challenging enough for them personally. One participant from the mindfulness group expressed frustration because she found her thoughts constantly wandering off during mindfulness meditation. Both participants of the integrative yoga class felt that they particularly benefited from a group exchange about stress in daily life.

*“You’re not so completely alone in the world, it’s not an exceptional problem, it’s something that a lot of people have.”*

(Participant 3—Integrative yoga)

Both participants of the Iyengar yoga class would have wanted advice and training on stress management, something their class did not entail. In contrast, both participants from the integrative yoga class, who received such content as part of their class, perceived it as particularly helpful. All instructors were perceived exclusively positively. They were described as good, professional, competent, helpful, and motivating.

Both participants from the Iyengar group claimed they did not learn coping mechanisms for stress in daily life, but found momentary relaxation when practicing yoga poses. One participant from the mindfulness group felt he had improved his stress management. The other participant from that group stated he now handled fear better in daily life. One participant from the integrative yoga group expressed that she had learned to handle stress better by better distancing herself from it.

An improved ability to handle stress was an expectation that was formulated by participants from all groups. The fears were that yoga or mindfulness would be uncomfortably alien and regular participation would be too stressful. This was despite the fact that all interviewees had prior experiences with yoga or mindfulness. All interviewees had resolved to practice yoga or mindfulness regularly in the future.

### 3.3. Harms

No physical or psychological harms or adverse effects related to the study interventions were reported. Yet, out of the participants who dropped out, some gave as a reason increased perceived stress due to the additional scheduling commitments of study participation. None of them reported a deterioration compared to their pre-study state. No SARS-CoV-2-transmissions were reported among study participants, instructors, or study personnel.

## 4. Discussion

The aim of this three-arm, randomized, controlled clinical trial was to compare the effects of integrative yoga, Iyengar yoga, and mindfulness training on participants with elevated stress levels and stress-related symptoms. All three interventions showed equally very large and stable effects in stress reduction and large effects in improvement of quality of life in the quantitative part of the study. There were no statistically significant group differences or indications of relevant synergistic effects of an “integrative” intervention combining mindfulness, physical exercises, and ethical/philosophical aspects of yoga over mindfulness or physical yoga exercise alone. The superiority of the integrative yoga approach in the sense of a Whole Medical System could not be demonstrated on the basis of the parameters examined during the study period. The observed effect sizes are consistent with those from a preliminary study of our research team [57], and with a systematic review by Della Valle et al. from 2020 which evaluated yoga interventions against inactive controls [104]. The relaxation procedures were studied in a form that largely corresponds to the usual application in clinical/psychological practice. We, therefore, assume that the results are generally transferable to clinical practice and prevention.

Major limitations of this study include: no blinding, a sample that was not representative of the overall population, a randomly selected sub-sample instead of a theoretical sample for the qualitative interviews, the onset of the COVID-19 pandemic during the study period, the resulting switch to an online format for the interventions, and a high dropout rate. An objective measurement of individual stress levels would have enriched the study. Measurements of cortisol levels or heart rate variability (HRV) were considered at a planning stage. Both methods allow objective conclusions about the activity of the autonomic nervous system (ANS) and the hypothalamic–pituitary–adrenocortical axis (HPA). Twenty-four-hour HRV was measured before and after the intervention in the first cohort of study participants. However, due to the COVID-19 pandemic, on-site visits were not possible, so too few data sets were available to perform an analysis.

Blinding was initially considered, but then deemed not fully feasible. It became apparent that too large a proportion of those interested in the study would have been able to independently infer their own group allocation. Despite advertising in public spaces, it appears that predominantly well-educated middle-aged women with an existing interest in mindfulness or yoga were recruited (this may also explain the low interest in the originally planned progressive muscle relaxation intervention.) The reasons for this could be higher stress levels in women and a higher likelihood of women seeking help for stress. A 2021 study on stress in the German population found that more women feel stressed occasionally or frequently (65% vs. 63%), and significantly more women reported seeking help in cases of severe stress (75% vs. 54%) [20]. This selection bias limits the transferability of the results to the general population. However, transferability to a population that is interested, and, thus, particularly amenable to the methods, can be discussed. Further studies evaluating stress reduction interventions including, e.g., low-income, racially diverse adults are warranted [105].

The occurrence of the COVID-19 pandemic during the study is a strong confounder with respect to subjective stress levels. The population average had a greatly increased stress level [106,107]. At the same time, this varied greatly at the individual level [107]. Given successful randomization, it can be assumed that all groups were equally affected. Therefore, the interpretation of group differences is not affected, but effect sizes from baseline are to an unknown extent. The change to an online format represents an important confounder of its own. The operationalization of the studied relaxation procedures was changed considerably as a result. There were large changes in setting (exercise environment, including background noise, space available, and distractions), whereas temporal exposure, instructional content, and movement patterns were little affected to unaffected.

For the qualitative part of the study, a theoretical sample would have been the methodologically better choice. Nevertheless, theoretical saturation was achieved. The interviews showed that only participants in the mindfulness and integrative intervention felt they had acquired better skills for dealing with stress in everyday life. Yet, perceived stress reduced equally in all intervention groups in the quantitative data.

Several studies indicate improved efficacy with respect to various outcomes when different aspects of yoga and meditation are combined in the sense of a Whole Medical System [108]. One possible interpretation of the very similar effects of all interventions examined in this study is that “time spent relaxing” is a relevant factor in stress reduction. The duration of exposure to each intervention was identical in all groups. Differences in physical engagement or learning mindfulness in the sense of the study hypotheses made no relevant impact. It is, therefore, conceivable that a dose–response relationship exists, which is not primarily dependent on the choice of a relaxation method. This would also fit with the observations of Cramer et al., who postulated that the effectiveness of different yoga styles was due to the personal preferences of the practitioners [55]. This thesis can possibly be extended to other relaxation methods. In this context, it is noted that the physical aspect of yoga did not play a major role in the participants’ perceptions. In relation to stress reduction, this factor may be less relevant. Time spent relaxing could, therefore, be a relevant factor in the effectiveness of relaxation techniques by itself.

Individual participants from all groups reported that the additional scheduling demands of study participation contributed to an increase in their stress experience. This led to the discontinuation of study participation for some. It is possible that continued study participation would still have had an impact on these participants. Nevertheless, it is questionable whether an additional appointment for relaxation is a viable option for all people with high subjective stress levels [57]. This observation does not necessarily allow conclusions to be drawn about the effectiveness of the methods studied. Rather, it should point to the societal problem of a high external stress load, which cannot be completely solved with improved methods of individual stress management.

## 5. Conclusions

All of the interventions studied were found to be comparably effective methods of stress reduction. There was no evidence of synergistic effects in stress reduction by combining yoga exercises with mindfulness or by using yoga as a Whole Medical System.

## Figures and Tables

**Table 1:** Eligibility criteria.

Inclusion Criteria	Exclusion Criteria
Age 18–65 years Subjectively perceived stress ≥ 4 out of 10 on a numeric analog scale (Numeric Rating-Scale, NRS 0–10, 0 = no stress at all to 10 = maximum stress) for ≥1 month At least 3 of the following 8 stress symptoms: sleep disorder, inappetence or increased appetite, shoulder neck tension/back pain, tension headache, concentration disorder, exhaustion, nervousness/irritability, stress-associated digestive problems	Pregnancy or breastfeeding Serious acute or chronic illness Serious mental illness Immobility/limitation for gymnastic exercises due to orthopedic, neurological, or other medical reason Participation in another study

**Table 2:** Baseline characteristics.

Characteristic	Total	Integrative Yoga	Iyengar Yoga	Mindfulness
Female (%)	91 (89.2%)	31 (88.6%)	28 (84.8%)	32 (94.1%)
Male (%)	11 (10.9%)	4 (11.4%)	5 (15.2%)	2 (5.9%)
Age (SD)	46.7 (11.5)	46.0 (11.5)	48.4 (10.6)	45.7 (12.5)
**Highest level of education**				
University (%)	73 (71.6%)	25 (71.4%)	24 (72.7%)	24 (70.6%)
Highschool (%)	13 (12.7%)	4 (11.4%)	4 (12.1%)	5 (14.7%)
Secondary school (%)	12 (11.8%)	4 (11.4%)	3 (9.1%)	5 (14.7%)
No graduation (%)	4 (3.9%)	2 (5.7%)	2 (6.1%)	0 (0.0%)
**Employment status**				
Full-time (%)	39 (38.2%)	16 (45.7%)	9 (27.3%)	14 (41.2%)
Part-time (%)	27 (26.4%)	7 (20.0%)	13 (39.4%)	7 (20.5%)
Student/training (%)	5 (4.9%)	1 (2.9%)	1 (3.0%)	3 (8.8%)
Unemployed (%)	1 (1.0%)	0 (0.0%)	0 (0.0%)	1 (2.9%)
Pension (%)	5 (4.9%)	2 (5.7%)	3 (9.1%)	0 (0.0%)
**Self-reported monthly income**				
<1000€ (%)	16 (15.7%)	8 (22.9%)	4 (12.1%)	4 (11.8%)
1001€–1500€ (%)	13 (12.7%)	5 (14.3%)	3 (9.1%)	5 (14.7%)
1501€–2000€ (%)	14 (13.7%)	5 (14.3%)	7 (21.2%)	2 (5.9%)
2001€–3000€ (%)	25 (24.5%)	10 (28.6%)	6 (18.2%)	9 (26.5%)
3001€–4000€ (%)	7 (6.9%)	2 (5.7%)	2 (6.1%)	3 (8.8%)
>4000€ (%)	4 (3.9%)	0 (0.0%)	4 (12.1%)	0 (0.0%)
Not stated	1 (1.0%)	1 (2.9%)	0 (0.0%)	0 (0.0%)
**Most common pre-existing conditions ^1^**				
Sleep disorder	29 (28.4%)	8 (22.9%)	12 (36.4%)	9 (26.5%)
Neck pain	28 (27.5%)	10 (28.6%)	10 (30.3%)	8 (23.5%)
Back pain	23 (22.5%)	5 (14.3%)	10 (30.3%)	8 (23.5%)
Digestive complaints	22 (21.6%)	9 (25.7%)	6 (18.2%)	7 (20.6%)
Tension headache	13 (12.7%)	4 (11.4%)	4 (12.1%)	5 (14.7%)
Hypertension	8 (7.8%)	6 (17.1%)	1 (3.0%)	1 (2.9%)
**Stress (PSS)**				
Score (SD)		33.91 (4.80)	33.39 (5.91)	34.44 (5.84)
**Burnout (MBI)**				
Cynicism (SD)		12.94 (8.79)	10.33 (6.90)	12.94 (6.96)
Exhaustion (SD)		18.83 (6.36)	19.52 (6.89)	20.53 (6.19)
Professional Efficacy (SD)		25.46 (6.22)	25.48 (6.88)	24.21 (7.06)
**Quality of Life (SF-36)**				
Mental Component Summary (SD)		36.79 (7.30)	38.86 (6.56)	37.94 (7.46)
Physical Component Summary (SD)		45.74 (6.06)	44.25 (6.35)	45.30 (7.22)
**Physical Wellbeing (B-LR)**				
Score (SD)		25.46 (7.72)	26.30 (8.38)	26.00 (8.81)
**Depression (HADS)**				
Anxiety (SD)		10.34 (2.91)	10.15 (3.46)	9.91 (3.53)
Depression (SD)		7.86 (3.79)	8.36 (3.80)	7.12 (4.00)
**Mindfulness (FMI)**				
Score (SD)		2.28 (0.48)	2.35 (0.50)	2.35 (0.37)
**Interoceptive body awareness (MAIA)**				
Noticing (SD)		3.23 (1.06)	3.21 (0.95)	3.33 (1.03)
Not-distracting (SD)		2.06 (0.91)	1.95 (1.00)	1.73 (0.65)
Not-worrying (SD)		2.06 (1.08)	2.24 (1.21)	2.15 (0.81)
Attention regulation (SD)		2.01 (1.00)	1.95 (1.05)	1.96 (0.93)
Emotional awareness (SD)		3.65 (0.92)	3.08 (1.12)	3.28 (1.02)
Self-regulation (SD)		1.99 (0.94)	1.75 (1.12)	1.87 (1.05)
Body listening (SD)		1.80 (0.97)	1.69 (1.38)	1.67 (1.13)
Trusting (SD)		2.51 (1.15)	2.33 (1.40)	2.42 (1.18)
**Self-regulation (SRI)**				
Score (SD)		3.35 (0.87)	3.36 (0.74)	3.58 (0.76)
**Spirituality (ASP)**				
Religious orientation (SD)		2.10 (0.95)	1.89 (0.91)	1.88 (0.93)
Search for wisdom (SD)		2.24 (0.72)	2.19 (0.65)	2.23 (0.70)
Conscious interactions (SD)		2.86 (0.55)	2.79 (0.81)	2.86 (0.67)
Transcendence conviction (SD)		1.83 (0.97)	1.68 (0.90)	1.72 (0.94)
**Mysticism (HMS)**				
Introvertive mysticism (SD)		1.74 (3.51)	3.18 (3.23)	2.74 (3.46)
Extrovertive mysticism (SD)		1.03 (2.29)	0.91 (2.47)	1.12 (2.88)
Interpretation (SD)		2.46 (3.23)	2.70 (3.57)	2.65 (3.67)
**Posttraumatic stress (PCL-5)**				
Score (SD)		26.77 (13.80)	26.03 (13.33)	27.00 (15.02)

^1^ A complete presentation of all pre-existing conditions can be found in the Supplementary Materials: Pre-existing conditions.

**Table 3:** Perceived Stress Scale (Primary Outcome—Mean ± SD).

	n	V0	V1	V2	ΔV0–V1	d V0–V1	ΔV0–V2	d V0–V2
**Integrative yoga**	35	33.91 ± 4.80	27.23 ± 6.77	26.03 ± 4.91	−6.69 ± 6.19	1.08	−7.89 ± 5.60	1.41
**Iyengar yoga**	33	33.39 ± 5.91	27.39 ± 6.98	23.88 ± 5.58	−6.00 ± 7.37	0.81	−9.52 ± 6.94	1.37
**Mindfulness**	34	34.44 ± 5.84	24.71 ± 6.67	23.62 ± 6.43	−9.74 ± 7.80	1.25	−10.82 ± 8.77	1.23
			**ΔV0–V1**			**ΔV0–V2**		
			**F**	**p**	**η^2^**	**F**	**p**	**η^2^**
**ANOVA**			2.621	0.078	0.050	1.439	0.242	0.028
			**ΔV0–V1**			**ΔV0–V2**		
**Post-hoc comparisons**			**t**	**p**	**η^2^**	**t**	**p**	**η^2^**
Integrative–Iyengar			−0.414	0.680	0.003	1.062	0.292	0.017
Integrative–Mindfulness			1.795	0.077	0.045	1.653	0.104	0.039
Iyengar–Mindfulness			2.016	0.048	0.057	0.678	0.500	0.007

**Table 4:** Participant information qualitative interviews.

Interview	Intervention	Participations	Gender	Course Period	Age
1	Iyengar yoga	11/12	female	January–April	48
2	Mindfulness training	11/12	male	January–April	50
3	Integrative yoga	8/12	female	January–April	58
4	Integrative yoga	10/12	female	January–April	47
5	Iyengar yoga	7/12	female	August–November	64
6	Mindfulness training	11/12	female	August–November	63

**Table 5:** Qualitative codes.

**Changes**
Everyday life
Self-care
Psychological changes
Somatic changes
Change as a process that takes time
**Course evaluation**
General evaluation
Educational share
Course instructor
**Other**
Hopes/Fears
Lockdown situation
Previous experience
Future

## Data Availability

Data from the study are available upon reasonable request.

## Supplementary Materials

The following supporting information can be downloaded at: https://www.mdpi.com/article/10.3390/jcm11195680/s1.
