# Supplementary material for: Stress Reduction by Yoga versus Mindfulness Training in Adults Suffering from Distress: A Three-Armed Randomized Controlled Trial including Qualitative Interviews (RELAX Study)
Source: J Clin Med. 2022 Sep 26;11(19):5680. doi: 10.3390/jcm11195680 (PMC9570550; doi:10.3390/jcm11195680)
Supplement: Supplementary file 1 [file jcm-11-05680-s001.zip › jcm-1915045-supplementary.pdf]

Journal of
Clinical Medicine

MDPI

Supplementary Material

Standard Operating Procedures **1**

Integrative Yoga Practice *1*

(Yama, Niyama, Asana, Pranayama, meditation, ethical/philosophical impulses) 1

Iyengar Yoga Practice *5*

Mindfulness Training *9*

Interview Guide – Qualitative Interviews **14**

Results – Randomized Controlled Trial **17**

Pre-existing Conditions *17*

Within Group Comparison – Overview effect size highlighted *17*

Within Group Comparison – Full *23*

Between group comparison (ANOVA) *29*

Diff V0 V1 29

Between group comparison (ANOVA) *33*

Diff V0 V2 33

Plot Practice Time *38*

Results – Qualitative Interviews **39**

1 Changes *39*
1.1 Change everyday life 39
General changes in everyday life 39
Daily practice 40
1.2 Change self care 41
1.3 Psychological changes 41
General mental changes 41
Dealing with stress 42
Serenity, More Relaxed, More Mindful 43
Dealing with fear 44
Emotional solution 45
1.4 Health changes 45
Sleep Change 46
Process that takes time 46
2 Course evaluation *46*
2.1 General evaluation 46
2.2 Educational part 47
2.3 Course instructor 47
2.4 Negative aspects of the courses 47
3 Other *48*
3.1 Hopes/Fears 48
3.2 Lockdown situation 49
3.3 Experience to date 49
3.4 Future 50

Standard Operating Procedures

Integrative Yoga Practice

(Yama, Niyama, Asana, Pranayama, meditation, ethical/philosophical impulses)

The intervention of integrative yoga practice consists of three phases that build on each other:

1. Low-threshold, simple exercise practice (4 weeks)
2. In-depth repetition with building practice (4 weeks)
3. In-depth repetition with building practice (4 weeks)

Exemplary sequence of a yoga class:

**Lesson 1-12**			
**Welcome & Arrival**	10 min	Welcome	2 min.
		Exchange: How are you doing? How was your exercise practice last week?	8 min.
**Theoretical input**	15 min.	Impulse lecture or worksheet	10 min.
		Exchange & conversation	5 min.
**Guided exercise practice**	60 min.	Supine position	10 min.
		Sitting exercises	5 min.
		Vinyasa	10 min.
		Standing exercises	10 min.
		Floor exercises	10 min.
		Relaxation	5 min.
		Pranayama & Silence Experience	10 min.
**Conclusion**	5 min.	Time for questions & exchange	3 min.
		Adoption	2 min.

Teaching unit	practical part	Input	Targets
**1. "What is stress?"**	• Introduction round & getting to know each other<br>• Motivation and needs of the participants<br>• Presentation of the course concept<br>• Introduction Exercise Practice 1<br>• Exchange & Questions<br>• Closing & Farewell	• PPT presentation on stress & ways to reduce stress.<br>• Discuss exercise practice 1 and guided practice.<br>• Introduction of the term *ahimsa* (non-harm) as a focus of practice and perception.	• Create a trusting atmosphere in the group<br>• "Form "working alliance<br>• Communicate the importance of stress for disease & health<br>• Stimulate reflection on stress perception and stress management strategies.<br>• Clarify conditions for good practice
**2. "How can I recognize stress?"**	• Welcome & sharing "How are you doing now? How has your home practice been?"<br>• in-depth repetition exercise practice 1<br>• Exchange & Questions<br>• Closing & Farewell	• Worksheet "How can you recognize stress?"<br>• Worksheet: Symptoms of tension and stress<br>• Introduction to different sitting postures	• Exchange & mutual inspiration in the group<br>• Perceive symptoms of stress on a physical, emotional and mental level & learn to reflect on one's own behavior<br>• Enable variations for relaxed sitting and thereby learn to take good care of yourself
**3. "What are we actually practicing?"**	• Welcome & sharing "How are you doing now? How has your home practice been?"<br>• in-depth repetition exercise practice 1<br>• Exchange & Questions<br>• Closing & Farewell	• Worksheet: "What are we actually practicing?" - Ashtanga yoga<br>• Worksheet: Exercise Triad	• Exchange & mutual inspiration in the group<br>• Presentation of Ashtanga Yoga with a focus on *ahimsa* (non-harm) as a practice and perception focus.
**4. "How can I manage stress?"**	• Welcome & sharing "How are you doing now? How has your home practice been?"<br>• in-depth repetition exercise practice 1<br>• Exchange & Questions<br>• Closing & Farewell	• Worksheet: differences between short-term relief and long-term change<br>• Worksheet: Rahe & Arthur ray path model	• Exchange & mutual inspiration in the group<br>• present different ways of stress reduction<br>• Recognize factors that lead to stressful experiences<br>• Present the influenceability of the stress reaction

**5. "How can I practice safely and successfully?"**	• Welcome & sharing "How are you doing now? How has your home practice been?"<br>• Introduction Exercise Practice 2<br>• Exchange & Questions<br>• Closing & Farewell	• Worksheet: Guidelines for safe & successful practice<br>• Worksheet: Introduce & explain the niyama *svadhyaya* (self-reflection)<br>• Discuss exercise practice 2 and practice together	• Exchange & mutual inspiration in the group<br>• Learning self-care through appropriate practice<br>• Learning to recognize and apply practice fields in yoga<br>• Develop & apply the "inner observer"<br>• Consolidate your own exercise practice
**6. "What are the stress management options in yoga?"**	• Welcome & sharing "How are you doing now? How has your home practice been?"<br>• in-depth repetition exercise practice 2<br>• Exchange & Questions<br>• Closing & Farewell	• Worksheet: Strategies for coping with stress and developing mindfulness.<br>• Worksheet: The Yogic Approach	• Exchange & mutual inspiration in the group<br>• Present yogic approach to stress reduction<br>• Teach mindfulness as a key to stress reduction and the importance of doing your own hands-on practice<br>• Select and apply a stress management strategy
**7. "Discover & use resources" 1**	• Welcome & sharing "How are you doing now? How has your home practice been?"<br>• in-depth repetition exercise practice 2<br>• Exchange & Questions<br>• Closing & Farewell	• Worksheet: Story of the woodcutter	• Exchange & mutual inspiration in the group<br>• Stimulate reflection on relaxation, rest & breaks as resources.
**8. "Discover & use resources" 2**	• Welcome & sharing "How are you doing now? How has your home practice been?"<br>• in-depth repetition exercise practice 2<br>• Exchange & Questions<br>• Closing & Farewell	• Worksheet: Guided relaxations as audio files (QR code)	• Exchange & mutual inspiration in the group<br>• offer further, low-threshold options for relaxation and stress reduction (audio file)

**9. "Practice serenity" 1**	• Welcome & sharing "How are you doing now? How has your home practice been?"<br>• Introduction Exercise Practice 3<br>• Exchange & Questions<br>• Closing & Farewell	• Worksheet: Introduce & explain the niyama *santosha* (serenity)<br>• Discuss exercise practice 3 and practice together	• Exchange & mutual inspiration in the group<br>• Teach serenity, mindfulness, and presence as a means of coping with stress<br>• Consolidate own exercise practice

**10. "Practice serenity" 2**	• Welcome & sharing "How are you doing now? How has your home practice been?"<br>• in-depth repetition exercise practice 3<br>• Exchange & Questions<br>• Closing & Farewell	• Worksheet: *Serenity Prayer* introduce & discuss	• Exchange & mutual inspiration in the group<br>• Teach serenity, mindfulness, and presence as a means of coping with stress
**11. "Applying mindfulness in everyday life" 1**	• Welcome & sharing "How are you doing now? How has your home practice been?"<br>• in-depth repetition exercise practice 3<br>• Exchange & Questions<br>• Closing & Farewell	• Worksheet: From symptom to cause (heyam, hetu, hanam, upaya)	• Exchange & mutual inspiration in the group<br>• Identify long-term changes and address them in everyday life
**12. "Applying mindfulness in everyday life" 2**	• Welcome & sharing "How are you doing now? How has your home practice been?"<br>• in-depth repetition exercise practice 3 summary & feedback<br>• Exchange & Questions<br>• Closing & Farewell	• drinking water together as a simple mindfulness exercise in everyday life<br>• Review & revise worksheet gallery<br>• Summary & Conclusion	• Exchange & mutual inspiration in the group<br>• Provide recognition that mindfulness can arise through all actions and be practiced in all activities, but requires practice<br>• Learning to pause and understand the idea of speedbreakers<br>• Transfer of the exercise experiences into everyday life

Iyengar Yoga Practice

Exemplary sequence of an Iyengar yoga class:

**Introduction**	10 min.	Welcome silent, upright sitting in Svastikasana (cross-legged sitting), or similar under detailed instruction and sounding of the syllable “om”<br>Urdhva Hastasana/ Parvatasana in Svastikansana (arms up/ fingers crossed cross-legged)<br>Tadasana (mountain pose) / Urdhva Hastasana/ Badhangulliasana
**Yoga postures**	70 min.	Uthita/Parsva Hasta Padasana (legs straddled/ foot, leg twisted)<br>Trikonasana (triangle pose)<br>VIrabhadrasana II second warrior<br>Parsva Konasana (side stretch)<br>Vriksasana (tree pose)<br>Svastikasana + parsva (upright twist)<br>Reverse postures (variations of Sarvangasana, shoulder stand)<br>Viparita Karani, pad under pelvis, calves on chair<br>legs in swastikasna on cushion
**Deep relaxation / Breath awareness**		Savasana (lying on back under detailed instructions)<br>Breath awareness, focus on observing the breath
**Conclusion**	10 min.	Discussion of the home exercise program<br>Final round and farewell

		Unit 1, 5 and 9<br>lateral standing postures	Unit 2 , 6 and 10<br>lateral / forward standing postures and seated postures	Unit 3, 7 and 11<br>lateral / backward standing postures and twist	Unit 4, 8 and 12<br>regenerative and inversion postures, prolonged abiding, pranayama
**Introduction**	6-10 min.	Welcome	Welcome	Welcome	Welcome
		sitting still, upright in Svastikasana (cross-legged seat), or similar, under detailed instructions and sounding the syllable "om	silent, upright sitting in Svastikasana, or similar, under detailed guidance and sounding the syllable "om	silent, upright sitting in Svastikasana, or similar, under detailed guidance and sounding the syllable "om	silent, upright sitting in Svastikasana, or similar, under detailed guidance and sounding the syllable "om
**Yoga poses**	approx. 70 min.	Urdhva Hastasana/ Parvatasana in Svastikansana (arms up/ fingers crossed cross-legged)	Urdhva Hastasana/ Parvatasana in Svastikansana	Urdhva Hastasana/ Parvatasana in Svastikansana	Supta Svastikasana with raised chest on cushion
			Adho Mukha Virasana (Child's Pose)	Adho Mukha Virasana	Supta Baddha Konasana with raised chest, thigh supported
			Adho Mukha Svanasana (Dog)	Adho Mukha Svanasana	Adho Mukha Svanasana on rope wall
			Uttanasana, holding elbow	Uttanasana, holding elbow	
		Tadasana (Mountain Pose) / Urdhva Hastasana/ Badhangulliasana	Tadasana / Urdhva Hastasana/Badhangulliasana	Tadasana / Urdhva Hastasana/Badhangulliasana	
		Uthita/Parsva Hasta Padasana (legs straddled/ foot, leg turned	Uthita/Parsva Hasta Padasana	Uthita/Parsva Hasta Padasana	
		Trikonasana,<br>(triangle posture)	Trikonasana,<br>(triangle posture)	Trikonasana<br>(triangle posture)	
		VIrabhadrasana II<br>second warrior	VIrabhadrasana II<br>second warrior	VIrabhadrasana II<br>second warrior	

		Parsva Konasana (side stretching)	Parsva Konasana (side stretching)	Parsva Konasana (side stretching)	
			Parsvottanasana, hands on hips	Virabhadrasana I (first warrior), both legs stretched	
			Parsvottanasana, hands on hips /on blocks	Virabhadrasana I (first warrior), full stance = front leg bent	
			Virasana, buttocks underlaid (Hero Seat)		
		Vriksasana (Tree Pose)		Bharadvajasana I with chair (swivel seat)	
		Svastikasana + parsva (turn erect)	Adho Mukha Svastikasana Forehead and elbows supported on chair (stretched forward cross-legged)		Sethu Bandha Sarvangasana with Pads (Variation of shoulder stand, but legs not higher than pelvis).
**Reverse postures (variations of Sarvangasana, shoulder stand)**		Viparita Karani, pad under pelvis, calves on chair	Viparita Karani, pad under pelvis, calves on chair	Viparita Karani, pad under pelvis, calves on chair	Viparita Karani, pad under pelvis, calves on chair
		Legs in swastikasna on cushion	Legs in swastikasna on cushion	Legs in swastikasna on cushion	Legs in swastikasna on cushion
**Deep relaxation/ Breath awareness**		Savasana (lying on the back under detailed instructions)	Savasana (lying on the back under detailed instructions)	Savasana (lying on the back under detailed instructions)	Savasana (lying on the back under detailed instructions)
		Breath awareness, focus	Breath awareness, focus on	Breath awareness, focus on	Breath awareness, focus

		on the observation of the breath	the observation of the breath	exhalation	on observing the breath, then consciously breathe evenly
**Conclusion**	approx. 10 min.	Discussion of the home exercise program	Discussion of the home exercise program	Discussion of the home exercise program	Discussion of the home exercise program
		Final round and farewell	Final round and farewell	Final round and farewell	Final round and farewell

Mindfulness Training

Exemplary structure of mindfulness training

**Session 1-12**			
**Introduction**	15 minutes	Welcome	3 minutes
		Practical part	12 minutes
**Theory**	15 minutes	Input	12 minutes
		Questions	3 minutes
**Mindfulness Practice**	45 minutes	Exercise / Meditation	35 minutes
		Reflection	10 minutes
**Conclusion**	15 minutes	Questions and exchange on home exercise and conclusions for upcoming home exercise	12 minutes
		Adoption	3 minutes

Input and group work alternate in their chronological sequence (see marking "A" and "B" respectively).

"B" in the table below on the contents).

	Practical part	Input	Mindfulness practice	Targets
1.	1.A Introductions.<br>Individual work: Collect different<br>Strategies in dealing with stress on cards	1.B Introduction of different levels of stress management;<br>Introduction to the Bodyscan (long form)	Bodyscan (long form)	Visualization of existing competences in the dealing with stress.<br>Knowledge of levels, at which stress management can be applied.<br>Body scan
**2.**	2.A Ball game with Half sentences - I guess in stress, when.../ I put myself under Stress, idem.../ when I am under stress, feel I.../	2.B Sources of stress and how the Stress response is triggered;<br>Introduction to the Concept of the Mindfulness	Bodyscan (long form)	Visualization personal stressors;<br>Meaning around Evaluation processes understand; mindfulness as mindfulness as Pillar of the Stress Management get to know

3.	3.A Pair exercise: How<br>do i stand by others?	3.B Introduction to the<br>Concept of the<br>self-compassion; the<br>Stress response<br>x-ray	Self-Compassion<br>pause	Holistic understanding for the<br>Stress response and<br>their consequences;<br>Self-compassion as<br>Pillar of the<br>Stress Management<br>get to know
4.	4.A Stress Exercise;<br>Reflection on<br>personal<br>thought patterns in the<br>Group	4.B Dealing with internal<br>Criticism:<br>stress-intensifying and<br>conducive thought patterns;<br>a mindful<br>Dealing with stressful<br>Find moments	Self-Compassion<br>pause	Dealing with<br>personal<br>Thinking patterns; Recognize<br>dysfunctional<br>patterns of thinking and their<br>Debilitation;<br>self-compassionate<br>Get to know handling
5.	5.A Couple interview with the inner driver to uncover personal target values; reflection on personal target values	5.B Dealing with expectations of us I: stress-reinforcing set points and their invalidation; introduction to metta meditation	Metta Meditation	Addressing stress exacerbating set points; moving toward beneficial set points; loving kindness as a pillar of stress management.<br>get to know
6.	6.B Individual work on good wishes	6.A Dealing with expectations of ourselves II: Introduction to good	personal metta meditation	invalidate dysfunctional expectations with an individual

		wishes for ourselves and differentiation from demands on ourselves<br>itself		selection of good wishes; individual sets of loving<br>Develop goodness
7.	7.B Individual Work for Compassionate Letter to Ourselves	7.A Dealing with Expectations of Ourselves III: Introduction to the Compassionate Letter to Ourselves	personal metta meditation	Strengthen self-acceptance; dysfunctional expectations through loving self-care.<br>invalidate
8.	8.A Individual work on difficult feelings	8.B Introduction to dealing with difficult feelings	Emotional exercise	mindful handling of feelings as a pillar of stress management<br>get to know
9.	9.B Individual work on inner parts	9.A Introduction to the concept of inner parts	Emotional exercise	Discovery and confrontation with stress-causing and stress-exacerbating inner parts; understanding of the good intentions of the inner part; understanding of the difficulties of getting into the<br>the share leads
10.	10.B Individual work on the inner parts	10.A<br>Stress-inducing	Selection of so far learned exercises/<br>Meditations	Stress-inducing<br>and

		and<br>stress aggravating<br>inner parts<br>get to know and make a<br>Find handling		stress exacerbating<br>inner parts better<br>and get to know their<br>Influence on the<br>Perception<br>understand; the shares<br>Give shape;<br>Possibilities in the<br>Find handling
11.	11.B Individual work to create an individual<br>Action Plan	11.A Introduction to mindful action	Selection of exercises/ meditations learned so far	Action plan for dealing with stressful situations
12.	12.B Individual work for good wishes to us<br>itself	12.A<br>Final Imagination	personal metta meditation	Review and perspectives; good<br>Wishes for us

-> Information about confidentiality and the course of the group training is given in the welcome of the 1st session.

Interview Guide – Qualitative Interviews

(Focus on how the course assigned to you works: Effect on stress perception, quality of life)

Entry question

- What experience did you have/do you have during the course you were assigned?

Changes and effects

Have you noticed any (concrete) changes in your life/daily routine while practicing mindfulness/yoga ?

- Changes in behavior in certain situations?

  (stressful, emotional, charged, challenging, etc.).

- Changes in how you treat yourself / your fellow human beings?

  Physical (Health) / Psychological (Mental) Changes?

  .. Perception, ...Emotional life, ...Mood/mood, ...Stress,

  ...sleep

  ...health/consumption/self-care self-esteem, ...acceptance, evaluation of one's own body.

- Changing your perspective on certain things
- What aspects of the course brought about these changes?

Possible stimuli: Have you noticed changes in your students? Have family/friends asked you about changes? (during the time)?

Course General:

a) Organizational

- Premises
- Teacher, program, procedure (What and how it was done?)

b) Personal contact

- What did you do well or like well
- What was not so good?
- How did you feel during / after the courses? (Always felt comfortable? -was a little uncomfortable, embarrassing-)
- Do you feel like it was helpful? (Stress perception)

  Which things did you already know, which aspects were new to you?

- What have you been able to take away? How have you benefited from the program so far?

  (How sustainable? What did you pick?)
- What disadvantages? Were there any other negative aspects that stood out during practice?
- Were there circumstances / situations in which it did you particularly good or bad?
- Do you want to continue practicing what you've learned?

c) What was your incentive to participate in this study?

- Previous experience?
- Expectations / fears / anxieties about it?
- Expectations fulfilled?

Would you recommend this stress reduction course to friends?

Conclusion

What do you take away for yourself for the future? (possibly: in relation to stressful situations)

Is there anything else that hasn't been addressed that you think I should still know?

Results – Randomized Controlled Trial

Pre-existing Conditions

	Integrative Yoga (n)	Iyengar Yoga (n)	Mindfulness (n)
Acne vulgaris	1	0	0
Anaemia	0	1	0
Anxiety disorder	3	1	2
Atrial fibrillation	1	0	0
Autoimmune gastritis	0	1	0
Back pain	5	10	8
Bronchial asthma	4	1	0
Bruxism	2	1	2
Colitis ulcerosa	0	2	0
Coronary heart disease	0	1	0
Chronic sinusitis	1	0	0
CREST-Syndrome	1	0	0
Digestive complaints	9	6	7
Exhaustion	2	3	3
Factor Vb deficiency	0	1	0
Fibromyalgia	2	0	0
Gonarthrosis	2	1	0
Hashimoto's thyroiditis	1	1	2
Tension headache	4	4	5
Herniated disc of the lumbar spine	1	1	0
Hypertension	6	1	1
Lichen planus	1	0	0
Lipedema	0	0	1
Lymphedema	0	0	1
Migraine	1	0	2

Mild depressive episode	3	3	2
Mixed connective tissue disease (MCTD)	0	0	1
Neck pain	10	10	8
Neurodermatitis	0	0	1
Non-essential tremor	0	0	1
Osteoporosis	0	0	1
Polyarthrosis	1	0	0
Psoriasis	0	1	1
Reflux esophagitis	0	1	2
Rheumatoid arthritis	0	1	0
SAPHO-Syndrome	0	0	1
Sleep disorder	8	12	9
Tinnitus	3	2	1
Urinary incontinence	0	0	1
Vertigo	0	0	1

Within Group Comparison – Overview effect size highlighted

**Effect size**	**Cohen's d**
Very small	0.01
Small	0.20
Medium	0.50
Large	0.80
Very large	1.20

		n	V0	V1	V2	ΔV0-V1	d V0-V1	ΔV0-V2	d V0-V2
**Perceived Stress (PSS)**									
Score (SD)	Integrative yoga	35	33.91 ± 4.80	27.23 ± 6.77	26.03 ± 4.91	-6.69 ± 6.19	1,08	-7.89 ± 5.60	1,41
	Iyengar yoga	33	33.39 ± 5.91	27.39 ± 6.98	23.88 ± 5.58	-6.00 ± 7.37	0,81	-9.52 ± 6.94	1,37
	Mindfulness	34	34.44 ± 5.84	24.71 ± 6.67	23.62 ± 6.43	-9.74 ± 7.80	1,25	-10.82 ± 8.77	1,23
**Burnout (MBI)**									
Cynicism (SD)	Integrative yoga	35	12.94 ± 8.79	10.17 ± 7.04	10.29 ± 7.54	-2.77 ± 6.36	0,44	-2.66 ± 6.60	0,40
	Iyengar yoga	33	10.33 ± 6.90	11.36 ± 7.66	10.64 ± 8.01	1.03 ± 6.48	0,16	0.30 ± 8.05	0,04
	Mindfulness	34	12.94 ± 6.96	9.88 ± 6.17	8.71 ± 6.81	-3.06 ± 8.98	0,34	-4.24 ± 9.49	0,45
Exhaustion (SD)	Integrative yoga	35	18.83 ± 6.36	14.91 ± 7.40	13.80 ± 6.87	-3.91 ± 6.05	0,65	-5.03 ± 6.28	0,80
	Iyengar yoga	33	19.52 ± 6.89	15.00 ± 7.91	12.42 ± 6.12	-4.52 ± 7.13	0,63	-7.09 ± 7.56	0,94
	Mindfulness	34	20.53 ± 6.19	13.03 ± 6.50	11.15 ± 5.18	-7.50 ± 8.24	0,91	-9.38 ± 8.31	1,13
Professional Efficacy (SD)	Integrative yoga	35	25.46 ± 6.22	26.89 ± 6.40	26.43 ± 6.33	1.43 ± 5.74	0,25	0.97 ± 6.33	0,15
	Iyengar yoga	33	25.48 ± 6.88	25.70 ± 6.76	26.73 ± 5.76	0.21 ± 6.82	0,03	1.24 ± 6.07	0,20
	Mindfulness	34	24.21 ± 7.06	27.41 ± 5.22	27.74 ± 5.82	3.21 ± 6.10	0,53	3.53 ± 6.54	0,54
**Quality of Life (SF-36)**									
Mental Component Summary (SD)	Integrative yoga	35	36.79 ± 7.30	44.34 ± 8.39	44.64 ± 6.69	7.55 ± 9.25	0,82	7.85 ± 6.92	1,13
	Iyengar yoga	33	38.86 ± 6.56	44.15 ± 9.19	44.77 ± 7.57	5.29 ± 8.99	0,59	5.92 ± 7.97	0,74
	Mindfulness	34	37.94 ± 7.46	42.03 ± 6.98	45.53 ± 6.92	4.09 ± 11.36	0,36	7.59 ± 9.41	0,81

Physical Component Summary (SD)	Integrative yoga	35	45.74 ± 6.06	45.36 ± 4.19	45.83 ± 4.01	-0.39 ± 6.89	0,06	0.09 ± 6.32	0,01
	Iyengar yoga	33	44.25 ± 6.35	44.98 ± 4.52	45.53 ± 4.72	0.73 ± 7.24	0,10	1.29 ± 5.99	0,21
	Mindfulness	34	45.30 ± 7.22	45.55 ± 4.28	45.23 ± 3.69	0.26 ± 7.00	0,04	-0.07 ± 6.98	0,01
**Physical Wellbeing (B-LR)**									
Score (SD)	Integrative yoga	35	25.46 ± 7.72	18.26 ± 10.08	15.57 ± 10.26	-7.20 ± 7.57	0,95	-9.89 ± 7.36	1,34
	Iyengar yoga	33	26.30 ± 8.38	17.45 ± 10.06	15.82 ± 8.97	-8.85 ± 8.21	1,08	-10.48 ± 8.17	1,28
	Mindfulness	34	26.00 ± 8.81	17.44 ± 9.68	14.12 ± 9.35	-8.56 ± 9.17	0,93	-11.88 ± 10.52	1,13
**Depression (HADS)**									
Anxiety (SD)	Integrative yoga	35	10.34 ± 2.91	7.17 ± 3.87	6.66 ± 3.43	-3.17 ± 3.87	0,82	-3.69 ± 4.16	0,89
	Iyengar yoga	33	10.15 ± 3.46	6.85 ± 3.91	6.48 ± 4.18	-3.30 ± 3.80	0,87	-3.67 ± 4.63	0,79
	Mindfulness	34	9.91 ± 3.53	7.09 ± 4.25	5.38 ± 3.72	-2.82 ± 4.61	0,61	-4.53 ± 4.94	0,92
Depression (SD)	Integrative yoga	35	7.86 ± 3.79	5.60 ± 4.67	4.94 ± 4.12	-2.26 ± 3.78	0,60	-2.91 ± 4.09	0,71
	Iyengar yoga	33	8.36 ± 3.80	6.67 ± 5.52	5.06 ± 3.95	-1.70 ± 4.08	0,42	-3.30 ± 3.16	1,05
	Mindfulness	34	7.12 ± 4.00	4.97 ± 4.12	4.06 ± 3.39	-2.15 ± 4.55	0,47	-3.06 ± 4.49	0,68
**Mindfulness (FMI)**									
Score (SD)	Integrative yoga	35	2.28 ± 0.48	2.62 ± 0.55	2.64 ± 0.46	0.35 ± 0.37	0,93	0.37 ± 0.50	0,73
	Iyengar yoga	33	2.35 ± 0.50	2.59 ± 0.55	2.79 ± 0.41	0.24 ± 0.47	0,52	0.43 ± 0.53	0,81
	Mindfulness	34	2.35 ± 0.37	2.71 ± 0.53	2.76 ± 0.42	0.36 ± 0.49	0,74	0.41 ± 0.56	0,73
**Interoceptive body awareness (MAIA)**									
Noticing (SD)	Integrative yoga	35	3.23 ± 1.06	3.36 ± 0.93	3.43 ± 0.84	0.14 ± 0.92	0,15	0.20 ± 0.92	0,22
	Iyengar yoga	33	3.21 ± 0.95	3.02 ± 1.18	3.28 ± 1.09	-0.20 ± 0.95	0,21	0.07 ± 0.93	0,07
	Mindfulness	34	3.33 ± 1.03	3.40 ± 1.25	3.56 ± 0.92	0.07 ± 1.13	0,06	0.23 ± 0.99	0,23
Not-distracting (SD)	Integrative yoga	35	2.06 ± 0.91	2.48 ± 0.81	2.43 ± 0.78	0.42 ± 0.74	0,57	0.37 ± 1.00	0,37
	Iyengar yoga	33	1.95 ± 1.00	2.64 ± 1.08	2.60 ± 0.74	0.69 ± 1.20	0,57	0.65 ± 1.15	0,56
	Mindfulness	34	1.73 ± 0.65	2.24 ± 1.11	2.00 ± 0.82	0.51 ± 1.31	0,39	0.27 ± 1.02	0,27
Not-worrying (SD)	Integrative yoga	35	2.06 ± 1.08	2.50 ± 0.83	2.53 ± 0.98	0.45 ± 0.91	0,49	0.48 ± 0.93	0,51
	Iyengar yoga	33	2.24 ± 1.21	2.82 ± 1.08	2.68 ± 0.89	0.58 ± 0.99	0,58	0.43 ± 0.96	0,45
	Mindfulness	34	2.15 ± 0.81	2.74 ± 0.93	2.56 ± 0.93	0.59 ± 1.06	0,56	0.41 ± 1.07	0,38
Attention regulation (SD)	Integrative yoga	35	2.01 ± 1.00	2.47 ± 1.00	2.56 ± 1.00	0.46 ± 0.80	0,57	0.55 ± 0.96	0,57
	Iyengar yoga	33	1.95 ± 1.05	2.35 ± 1.07	2.41 ± 1.08	0.39 ± 0.89	0,44	0.46 ± 0.99	0,46

	Mindfulness	34	1.96 ± 0.93	2.79 ± 1.07	2.62 ± 1.12	0.83 ± 1.19	0,70	0.66 ± 1.32	0,50
Emotional awareness (SD)	Integrative yoga	35	3.65 ± 0.92	3.66 ± 0.82	3.47 ± 1.14	0.02 ± 0.75	0,02	-0.17 ± 1.13	0,15
	Iyengar yoga	33	3.08 ± 1.12	3.04 ± 1.22	3.03 ± 1.29	-0.04 ± 0.86	0,05	-0.05 ± 0.98	0,06
	Mindfulness	34	3.28 ± 1.02	3.74 ± 0.94	3.63 ± 0.94	0.46 ± 1.13	0,41	0.35 ± 1.08	0,33
Self-regulation (SD)	Integrative yoga	35	1.99 ± 0.94	2.87 ± 1.04	2.76 ± 1.05	0.89 ± 0.99	0,89	0.78 ± 1.05	0,74
	Iyengar yoga	33	1.75 ± 1.12	2.30 ± 1.18	2.72 ± 0.97	0.55 ± 1.05	0,52	0.97 ± 1.06	0,91
	Mindfulness	34	1.87 ± 1.05	2.96 ± 1.25	2.86 ± 1.15	1.10 ± 1.24	0,88	0.99 ± 1.35	0,74
Body listening (SD)	Integrative yoga	35	1.80 ± 0.97	2.42 ± 1.06	2.66 ± 1.33	0.62 ± 1.15	0,54	0.86 ± 1.34	0,64
	Iyengar yoga	33	1.69 ± 1.38	2.28 ± 1.12	2.20 ± 1.27	0.60 ± 0.95	0,63	0.52 ± 1.21	0,42
	Mindfulness	34	1.67 ± 1.13	2.68 ± 1.34	2.65 ± 1.31	1.01 ± 1.48	0,68	0.98 ± 1.37	0,72
Trusting (SD)	Integrative yoga	35	2.51 ± 1.15	2.90 ± 0.97	3.16 ± 1.22	0.38 ± 0.76	0,50	0.65 ± 1.07	0,61
	Iyengar yoga	33	2.33 ± 1.40	2.70 ± 1.31	3.13 ± 1.24	0.36 ± 1.25	0,29	0.80 ± 1.29	0,62
	Mindfulness	34	2.42 ± 1.18	3.21 ± 1.17	3.53 ± 1.17	0.78 ± 0.95	0,82	1.11 ± 1.04	1,06
**Self-regulation (SRI)**									
Score (SD)	Integrative yoga	35	3.35 ± 0.87	3.88 ± 0.93	3.84 ± 0.91	0.53 ± 0.86	0,62	0.50 ± 0.96	0,52
	Iyengar yoga	33	3.36 ± 0.74	3.74 ± 1.06	3.85 ± 0.92	0.38 ± 0.93	0,40	0.49 ± 0.87	0,56
	Mindfulness	34	3.58 ± 0.76	4.06 ± 0.85	3.97 ± 0.79	0.49 ± 0.77	0,63	0.40 ± 0.75	0,52
**Spirituality (ASP)**									
Religious orientation (SD)	Integrative yoga	35	2.10 ± 0.95	2.13 ± 0.99	2.06 ± 0.93	0.03 ± 0.55	0,05	-0.05 ± 0.59	0,08
	Iyengar yoga	33	1.89 ± 0.91	1.77 ± 0.95	2.04 ± 0.85	-0.11 ± 0.38	0,30	0.15 ± 0.52	0,30
	Mindfulness	34	1.88 ± 0.93	2.02 ± 1.04	1.99 ± 0.95	0.14 ± 0.47	0,29	0.11 ± 0.45	0,25
Search for wisdom (SD)	Integrative yoga	35	2.24 ± 0.72	2.45 ± 0.69	2.48 ± 0.73	0.21 ± 0.46	0,45	0.24 ± 0.59	0,40
	Iyengar yoga	33	2.19 ± 0.65	2.26 ± 0.80	2.33 ± 0.70	0.07 ± 0.67	0,11	0.14 ± 0.66	0,22
	Mindfulness	34	2.23 ± 0.70	2.68 ± 0.68	2.36 ± 0.64	0.45 ± 0.66	0,69	0.13 ± 0.62	0,22
Conscious interactions (SD)	Integrative yoga	35	2.86 ± 0.55	3.03 ± 0.55	2.81 ± 0.71	0.17 ± 0.49	0,35	-0.04 ± 0.55	0,08
	Iyengar yoga	33	2.79 ± 0.81	2.78 ± 0.73	2.87 ± 0.62	-0.01 ± 0.61	0,02	0.08 ± 0.73	0,10
	Mindfulness	34	2.86 ± 0.67	2.94 ± 0.55	2.89 ± 0.65	0.08 ± 0.59	0,14	0.03 ± 0.72	0,05
Transcendence conviction (SD)	Integrative yoga	35	1.83 ± 0.97	1.93 ± 0.83	2.06 ± 0.95	0.10 ± 0.57	0,17	0.24 ± 0.71	0,33
	Iyengar yoga	33	1.68 ± 0.90	1.76 ± 0.91	1.82 ± 0.83	0.08 ± 0.77	0,10	0.14 ± 0.73	0,19
	Mindfulness	34	1.72 ± 0.94	1.85 ± 1.01	1.85 ± 0.96	0.13 ± 0.56	0,22	0.13 ± 0.49	0,27

**Mysticism (HMS)**									
Introvertive mysticism (SD)	Integrative yoga	35	1.74 ± 3.51	1.34 ± 4.24	1.77 ± 4.29	-0.40 ± 3.34	0,12	0.03 ± 3.91	0,01
	Iyengar yoga	33	3.18 ± 3.23	2.61 ± 3.88	3.42 ± 3.49	-0.58 ± 3.96	0,15	0.24 ± 2.84	0,09
	Mindfulness	34	2.74 ± 3.46	2.41 ± 3.73	3.00 ± 3.24	-0.32 ± 2.87	0,11	0.26 ± 3.51	0,08
Extrovertive mysticism (SD)	Integrative yoga	35	1.03 ± 2.29	0.40 ± 3.03	0.94 ± 3.02	-0.63 ± 2.49	0,25	-0.09 ± 2.59	0,03
	Iyengar yoga	33	0.91 ± 2.47	0.79 ± 2.51	1.67 ± 2.53	-0.12 ± 2.33	0,05	0.76 ± 2.36	0,32
	Mindfulness	34	1.12 ± 2.88	0.76 ± 2.81	1.50 ± 2.60	-0.35 ± 2.31	0,15	0.38 ± 2.75	0,14
Interpretation (SD)	Integrative yoga	35	2.46 ± 3.23	1.43 ± 3.81	1.97 ± 4.18	-1.03 ± 2.85	0,36	-0.49 ± 3.54	0,14
	Iyengar yoga	33	2.70 ± 3.57	2.58 ± 3.56	3.06 ± 3.24	-0.12 ± 2.76	0,04	0.36 ± 2.98	0,12
	Mindfulness	34	2.65 ± 3.67	2.38 ± 3.77	2.35 ± 3.73	-0.26 ± 2.51	0,11	-0.29 ± 3.76	0,08
**Posttraumatic stress (PCL-5)**									
Score (SD)	Integrative yoga	35	26.77 ± 13.80	17.71 ± 13.27	18.74 ± 12.90	-9.06 ± 16.02	0,57	-8.03 ± 16.33	0,49
	Iyengar yoga	33	26.03 ± 13.33	20.18 ± 15.51	18.18 ± 13.58	-5.85 ± 16.13	0,36	-7.85 ± 14.06	0,56
	Mindfulness	34	27.00 ± 15.02	18.59 ± 12.99	14.65 ± 8.98	-8.41 ± 17.92	0,47	-12.35 ± 16.90	0,73

Within Group Comparison – Full

Questionnaire	Interv	*n*	Diff V0V1 M	SD	t	p	d
PSS	Integrative Yoga	*35*	-6,69	6,19	-6,39	0,00	1,08
	Iyengar Yoga	*33*	-6,00	7,37	-4,68	0,00	0,81
	Mindfulness Training	*34*	-9,74	7,80	-7,28	0,00	1,25
ASPConscious	Integrative Yoga	*35*	0,17	0,49	2,07	0,05	0,35
	Iyengar Yoga	*33*	-0,01	0,61	-0,10	0,92	0,02
	Mindfulness Training	*34*	0,08	0,59	0,82	0,42	0,14
ASPReligiousOrient	Integrative Yoga	*35*	0,03	0,55	0,27	0,79	0,05
	Iyengar Yoga	*33*	-0,11	0,38	-1,72	0,10	0,30
	Mindfulness Training	*34*	0,14	0,47	1,70	0,10	0,29
ASPSearchWisdom	Integrative Yoga	*35*	0,21	0,46	2,65	0,01	0,45
	Iyengar Yoga	*33*	0,07	0,67	0,63	0,54	0,11
	Mindfulness Training	*34*	0,45	0,66	4,00	0,00	0,69
ASPTranscendence	Integrative Yoga	*35*	0,10	0,57	1,03	0,31	0,17
	Iyengar Yoga	*33*	0,08	0,77	0,56	0,58	0,10
	Mindfulness Training	*34*	0,13	0,56	1,31	0,20	0,22
BLR	Integrative Yoga	*35*	-7,20	7,57	-5,63	0,00	0,95
	Iyengar Yoga	*33*	-8,85	8,21	-6,19	0,00	1,08
	Mindfulness Training	*34*	-8,56	9,17	-5,44	0,00	0,93
BLR1	Integrative Yoga	*35*	-6,57	9,61	-4,05	0,00	0,68
	Iyengar Yoga	*33*	-6,67	9,17	-4,17	0,00	0,73
	Mindfulness Training	*34*	-7,59	10,55	-4,20	0,00	0,72
FFA	Integrative Yoga	*35*	0,35	0,37	5,48	0,00	0,93
	Iyengar Yoga	*33*	0,24	0,47	2,97	0,01	0,52
	Mindfulness Training	*34*	0,36	0,49	4,30	0,00	0,74
HADSA	Integrative Yoga	*35*	-3,17	3,87	-4,85	0,00	0,82

	**Iyengar Yoga**	*33*	-3,30	3,80	-4,99	0,00	0,87
	**Mindfulness Training**	*34*	-2,82	4,61	-3,57	0,00	0,61
**HADSD**	**Integrative Yoga**	*35*	-2,26	3,78	-3,53	0,00	0,60
	**Iyengar Yoga**	*33*	-1,70	4,08	-2,39	0,02	0,42
	**Mindfulness Training**	*34*	-2,15	4,55	-2,75	0,01	0,47
**MAIAAufmerkRegula**	**Integrative Yoga**	*35*	0,46	0,80	3,40	0,00	0,57
	**Iyengar Yoga**	*33*	0,39	0,89	2,54	0,02	0,44
	**Mindfulness Training**	*34*	0,83	1,19	4,08	0,00	0,70
**MAIABemerken**	**Integrative Yoga**	*35*	0,14	0,92	0,87	0,39	0,15
	**Iyengar Yoga**	*33*	-0,20	0,95	-1,19	0,24	0,21
	**Mindfulness Training**	*34*	0,07	1,13	0,34	0,73	0,06
**MAIAEmotGewahr**	**Integrative Yoga**	*35*	0,02	0,75	0,13	0,89	0,02
	**Iyengar Yoga**	*33*	-0,04	0,86	-0,28	0,78	0,05
	**Mindfulness Training**	*34*	0,46	1,13	2,40	0,02	0,41
**MAIALeibHören**	**Integrative Yoga**	*35*	0,62	1,15	3,19	0,00	0,54
	**Iyengar Yoga**	*33*	0,60	0,95	3,61	0,00	0,63
	**Mindfulness Training**	*34*	1,01	1,48	3,99	0,00	0,68
**MAIANichtAblenken**	**Integrative Yoga**	*35*	0,42	0,74	3,36	0,00	0,57
	**Iyengar Yoga**	*33*	0,69	1,20	3,30	0,00	0,57
	**Mindfulness Training**	*34*	0,51	1,31	2,27	0,03	0,39
**MAIANichtSorgen**	**Integrative Yoga**	*35*	0,45	0,91	2,92	0,01	0,49
	**Iyengar Yoga**	*33*	0,58	0,99	3,35	0,00	0,58
	**Mindfulness Training**	*34*	0,59	1,06	3,24	0,00	0,56
**MAIASelbstregula**	**Integrative Yoga**	*35*	0,89	0,99	5,28	0,00	0,89
	**Iyengar Yoga**	*33*	0,55	1,05	2,98	0,01	0,52
	**Mindfulness Training**	*34*	1,10	1,24	5,16	0,00	0,88
**MAIAVertrauen**	**Integrative Yoga**	*35*	0,38	0,76	2,97	0,01	0,50
	**Iyengar Yoga**	*33*	0,36	1,25	1,67	0,11	0,29
	**Mindfulness Training**	*34*	0,78	0,95	4,80	0,00	0,82
**MBICY**	**Integrative Yoga**	*35*	-2,77	6,36	-2,58	0,01	0,44

	Iyengar Yoga	*33*	1,03	6,48	0,91	0,37	0,16
	Mindfulness Training	*34*	-3,06	8,98	-1,99	0,06	0,34
MBIEX	Integrative Yoga	*35*	-3,91	6,05	-3,83	0,00	0,65
	Iyengar Yoga	*33*	-4,52	7,13	-3,64	0,00	0,63
	Mindfulness Training	*34*	-7,50	8,24	-5,31	0,00	0,91
MBIPE	Integrative Yoga	*35*	1,43	5,74	1,47	0,15	0,25
	Iyengar Yoga	*33*	0,21	6,82	0,18	0,86	0,03
	Mindfulness Training	*34*	3,21	6,10	3,06	0,00	0,53
MyIntrovert	Integrative Yoga	*35*	-0,40	3,34	-0,71	0,48	0,12
	Iyengar Yoga	*33*	-0,58	3,96	-0,84	0,41	0,15
	Mindfulness Training	*34*	-0,32	2,87	-0,66	0,52	0,11
MyExtrovert	Integrative Yoga	*35*	-0,63	2,49	-1,50	0,14	0,25
	Iyengar Yoga	*33*	-0,12	2,33	-0,30	0,77	0,05
	Mindfulness Training	*34*	-0,35	2,31	-0,89	0,38	0,15
MyInterpret	Integrative Yoga	*35*	-1,03	2,85	-2,13	0,04	0,36
	Iyengar Yoga	*33*	-0,12	2,76	-0,25	0,80	0,04
	Mindfulness Training	*34*	-0,26	2,51	-0,61	0,54	0,11
PCL	Integrative Yoga	*35*	-9,06	16,02	-3,34	0,00	0,57
	Iyengar Yoga	*33*	-5,85	16,13	-2,08	0,05	0,36
	Mindfulness Training	*34*	-8,41	17,92	-2,74	0,01	0,47
SF36Ment	Integrative Yoga	*35*	7,55	9,25	4,83	0,00	0,82
	Iyengar Yoga	*33*	5,29	8,99	3,38	0,00	0,59
	Mindfulness Training	*34*	4,09	11,36	2,10	0,04	0,36
SF36Phys	Integrative Yoga	*35*	-0,39	6,89	-0,33	0,74	0,06
	Iyengar Yoga	*33*	0,73	7,24	0,58	0,57	0,10
	Mindfulness Training	*34*	0,26	7,00	0,21	0,83	0,04
SR	Integrative Yoga	*35*	0,53	0,86	3,64	0,00	0,62
	Iyengar Yoga	*33*	0,38	0,93	2,32	0,03	0,40
	Mindfulness Training	*34*	0,49	0,77	3,68	0,00	0,63

Questionnaire	Interv	*n*	Diff V0V2 M	SD	t	p	d
PSS	Integrative Yoga	*35*	-7,89	5,60	-8,33	0,00	1,41
	Iyengar Yoga	*33*	-9,52	6,94	-7,88	0,00	1,37
	Mindfulness Training	*34*	-10,82	8,77	-7,20	0,00	1,23
ASPConscious	Integrative Yoga	*35*	-0,04	0,55	-0,46	0,65	0,08
	Iyengar Yoga	*33*	0,08	0,73	0,60	0,55	0,10
	Mindfulness Training	*34*	0,03	0,72	0,28	0,78	0,05
ASPReligiousOrient	Integrative Yoga	*35*	-0,05	0,59	-0,45	0,65	0,08
	Iyengar Yoga	*33*	0,15	0,52	1,72	0,09	0,30
	Mindfulness Training	*34*	0,11	0,45	1,48	0,15	0,25
ASPSearchWisdom	Integrative Yoga	*35*	0,24	0,59	2,38	0,02	0,40
	Iyengar Yoga	*33*	0,14	0,66	1,25	0,22	0,22
	Mindfulness Training	*34*	0,13	0,62	1,27	0,21	0,22
ASPTranscendence	Integrative Yoga	*35*	0,24	0,71	1,95	0,06	0,33
	Iyengar Yoga	*33*	0,14	0,73	1,08	0,29	0,19
	Mindfulness Training	*34*	0,13	0,49	1,58	0,12	0,27
BLR	Integrative Yoga	*35*	-9,89	7,36	-7,95	0,00	1,34
	Iyengar Yoga	*33*	-10,48	8,17	-7,38	0,00	1,28
	Mindfulness Training	*34*	-11,88	10,52	-6,58	0,00	1,13
BLR1	Integrative Yoga	*35*	-9,94	8,79	-6,69	0,00	1,13
	Iyengar Yoga	*33*	-8,67	8,89	-5,60	0,00	0,97
	Mindfulness Training	*34*	-10,29	11,11	-5,40	0,00	0,93
FFA	Integrative Yoga	*35*	0,37	0,50	4,34	0,00	0,73
	Iyengar Yoga	*33*	0,43	0,53	4,68	0,00	0,81
	Mindfulness Training	*34*	0,41	0,56	4,25	0,00	0,73
HADSA	Integrative Yoga	*35*	-3,69	4,16	-5,24	0,00	0,89
	Iyengar Yoga	*33*	-3,67	4,63	-4,54	0,00	0,79
	Mindfulness Training	*34*	-4,53	4,94	-5,34	0,00	0,92

**HADSD**	**Integrative Yoga**	*35*	-2,91	4,09	-4,22	0,00	0,71
	**Iyengar Yoga**	*33*	-3,30	3,16	-6,01	0,00	1,05
	**Mindfulness Training**	*34*	-3,06	4,49	-3,97	0,00	0,68
**MAIAAufmerkRegula**	**Integrative Yoga**	*35*	0,55	0,96	3,39	0,00	0,57
	**Iyengar Yoga**	*33*	0,46	0,99	2,66	0,01	0,46
	**Mindfulness Training**	*34*	0,66	1,32	2,92	0,01	0,50
**MAIABemerken**	**Integrative Yoga**	*35*	0,20	0,92	1,28	0,21	0,22
	**Iyengar Yoga**	*33*	0,07	0,93	0,42	0,68	0,07
	**Mindfulness Training**	*34*	0,23	0,99	1,35	0,19	0,23
**MAIAEmotGewahr**	**Integrative Yoga**	*35*	-0,17	1,13	-0,90	0,38	0,15
	**Iyengar Yoga**	*33*	-0,05	0,98	-0,32	0,75	0,06
	**Mindfulness Training**	*34*	0,35	1,08	1,91	0,06	0,33
**MAIALeibHören**	**Integrative Yoga**	*35*	0,86	1,34	3,77	0,00	0,64
	**Iyengar Yoga**	*33*	0,52	1,21	2,44	0,02	0,42
	**Mindfulness Training**	*34*	0,98	1,37	4,19	0,00	0,72
**MAIANichtAblenken**	**Integrative Yoga**	*35*	0,37	1,00	2,21	0,03	0,37
	**Iyengar Yoga**	*33*	0,65	1,15	3,23	0,00	0,56
	**Mindfulness Training**	*34*	0,27	1,02	1,57	0,12	0,27
**MAIANichtSorgen**	**Integrative Yoga**	*35*	0,48	0,93	3,02	0,00	0,51
	**Iyengar Yoga**	*33*	0,43	0,96	2,59	0,01	0,45
	**Mindfulness Training**	*34*	0,41	1,07	2,24	0,03	0,38
**MAIASelbstregula**	**Integrative Yoga**	*35*	0,78	1,05	4,38	0,00	0,74
	**Iyengar Yoga**	*33*	0,97	1,06	5,24	0,00	0,91
	**Mindfulness Training**	*34*	0,99	1,35	4,29	0,00	0,74
**MAIAVertrauen**	**Integrative Yoga**	*35*	0,65	1,07	3,58	0,00	0,61
	**Iyengar Yoga**	*33*	0,80	1,29	3,56	0,00	0,62
	**Mindfulness Training**	*34*	1,11	1,04	6,21	0,00	1,06
**MBICY**	**Integrative Yoga**	*35*	-2,66	6,60	-2,38	0,02	0,40
	**Iyengar Yoga**	*33*	0,30	8,05	0,22	0,83	0,04
	**Mindfulness Training**	*34*	-4,24	9,49	-2,60	0,01	0,45

**MBIEX**	**Integrative Yoga**	*35*	-5,03	6,28	-4,73	0,00	0,80
	**Iyengar Yoga**	*33*	-7,09	7,56	-5,39	0,00	0,94
	**Mindfulness Training**	*34*	-9,38	8,31	-6,58	0,00	1,13
**MBIPE**	**Integrative Yoga**	*35*	0,97	6,33	0,91	0,37	0,15
	**Iyengar Yoga**	*33*	1,24	6,07	1,18	0,25	0,20
	**Mindfulness Training**	*34*	3,53	6,54	3,15	0,00	0,54
**MyIntrovert**	**Integrative Yoga**	*35*	0,03	3,91	0,04	0,97	0,01
	**Iyengar Yoga**	*33*	0,24	2,84	0,49	0,63	0,09
	**Mindfulness Training**	*34*	0,26	3,51	0,44	0,66	0,08
**MyExtrovert**	**Integrative Yoga**	*35*	-0,09	2,59	-0,20	0,85	0,03
	**Iyengar Yoga**	*33*	0,76	2,36	1,84	0,07	0,32
	**Mindfulness Training**	*34*	0,38	2,75	0,81	0,42	0,14
**MyInterpret**	**Integrative Yoga**	*35*	-0,49	3,54	-0,81	0,42	0,14
	**Iyengar Yoga**	*33*	0,36	2,98	0,70	0,49	0,12
	**Mindfulness Training**	*34*	-0,29	3,76	-0,46	0,65	0,08
**PCL**	**Integrative Yoga**	*35*	-8,03	16,33	-2,91	0,01	0,49
	**Iyengar Yoga**	*33*	-7,85	14,06	-3,21	0,00	0,56
	**Mindfulness Training**	*34*	-12,35	16,90	-4,26	0,00	0,73
**SF36Ment**	**Integrative Yoga**	*35*	7,85	6,92	6,71	0,00	1,13
	**Iyengar Yoga**	*33*	5,92	7,97	4,26	0,00	0,74
	**Mindfulness Training**	*34*	7,59	9,41	4,70	0,00	0,81
**SF36Phys**	**Integrative Yoga**	*35*	0,09	6,32	0,08	0,93	0,01
	**Iyengar Yoga**	*33*	1,29	5,99	1,23	0,23	0,21
	**Mindfulness Training**	*34*	-0,07	6,98	-0,06	0,96	0,01
**SR**	**Integrative Yoga**	*35*	0,50	0,96	3,07	0,00	0,52
	**Iyengar Yoga**	*33*	0,49	0,87	3,24	0,00	0,56
	**Mindfulness Training**	*34*	0,40	0,75	3,05	0,00	0,52

Between group comparison (ANOVA)

Diff V0 V1

Questionnaire	Diff V0 V1 ANOVA F	p	eta2	Post-hoc Comparisons comp	t	p	eta2
PSS	2,621	0,078	0,050	Integr-Iyengar	-0,414	0,680	0,003
				Integr-Mind	1,795	0,077	0,045
				Iyengar-Mind	2,016	0,048	0,057
ASPConscious	0,880	0,418	0,017	Integr-Iyengar	1,353	0,181	0,027
				Integr-Mind	0,673	0,504	0,007
				Iyengar-Mind	-0,638	0,526	0,006
ASPReligiousOrient	2,364	0,099	0,046	Integr-Iyengar	1,224	0,226	0,021
				Integr-Mind	-0,908	0,367	0,012
				Iyengar-Mind	-2,404	0,019	0,079
ASPSearchWisdom	3,349	0,039	0,063	Integr-Iyengar	0,953	0,345	0,013
				Integr-Mind	-1,762	0,083	0,043
				Iyengar-Mind	-2,313	0,024	0,074
ASPTranscendence	0,050	0,952	0,001	Integr-Iyengar	0,146	0,884	0,000
				Integr-Mind	-0,184	0,855	0,000
				Iyengar-Mind	-0,299	0,766	0,001
BLR	0,383	0,683	0,008	Integr-Iyengar	0,859	0,393	0,011
				Integr-Mind	0,670	0,505	0,007
				Iyengar-Mind	-0,136	0,892	0,000
BLR1	0,112	0,894	0,002	Integr-Iyengar	0,042	0,967	0,000
				Integr-Mind	0,418	0,677	0,003
				Iyengar-Mind	0,382	0,704	0,002
FFA	0,738	0,481	0,015	Integr-Iyengar	1,038	0,303	0,016
				Integr-Mind	-0,137	0,892	0,000

				Iyengar-Mind	-1,037	0,303	0,016
HADSA	0,122	0,885	0,002	Integr-Iyengar	0,141	0,888	0,000
				Integr-Mind	-0,339	0,736	0,002
				Iyengar-Mind	-0,465	0,643	0,003
HADSD	0,172	0,842	0,003	Integr-Iyengar	-0,586	0,560	0,005
				Integr-Mind	-0,109	0,913	0,000
				Iyengar-Mind	0,426	0,671	0,003
MAIAAufmerkRegula	2,008	0,140	0,039	Integr-Iyengar	0,308	0,759	0,001
				Integr-Mind	-1,535	0,130	0,033
				Iyengar-Mind	-1,710	0,092	0,041
MAIABemerken	1,027	0,362	0,020	Integr-Iyengar	1,464	0,148	0,031
				Integr-Mind	0,280	0,780	0,001
				Iyengar-Mind	-1,033	0,306	0,016
MAIAEmotGewahr	3,019	0,053	0,057	Integr-Iyengar	0,302	0,763	0,001
				Integr-Mind	-1,930	0,059	0,052
				Iyengar-Mind	-2,066	0,043	0,059
MAIALeibHören	1,248	0,292	0,025	Integr-Iyengar	0,091	0,928	0,000
				Integr-Mind	-1,225	0,225	0,021
				Iyengar-Mind	-1,369	0,176	0,027
MAIANichtAblenken	0,513	0,600	0,010	Integr-Iyengar	-1,104	0,275	0,018
				Integr-Mind	-0,353	0,726	0,002
				Iyengar-Mind	0,578	0,565	0,005
MAIANichtSorgen	0,216	0,806	0,004	Integr-Iyengar	-0,556	0,580	0,005
				Integr-Mind	-0,592	0,556	0,005
				Iyengar-Mind	-0,050	0,960	0,000
MAIASelbstregula	2,135	0,124	0,041	Integr-Iyengar	1,371	0,175	0,027
				Integr-Mind	-0,775	0,441	0,009
				Iyengar-Mind	-1,963	0,054	0,054
MAIAVertrauen	1,907	0,154	0,037	Integr-Iyengar	0,068	0,946	0,000
				Integr-Mind	-1,941	0,057	0,052

				Iyengar-Mind	-1,543	0,128	0,035
MBICY	3,206	0,045	0,061	Integr-Iyengar	-2,440	0,017	0,081
				Integr-Mind	0,153	0,879	0,000
				Iyengar-Mind	2,142	0,036	0,064
MBIEX	2,442	0,092	0,047	Integr-Iyengar	0,374	0,710	0,002
				Integr-Mind	2,057	0,044	0,058
				Iyengar-Mind	1,587	0,117	0,036
MBIPE	1,965	0,146	0,038	Integr-Iyengar	0,794	0,430	0,009
				Integr-Mind	-1,246	0,217	0,022
				Iyengar-Mind	-1,892	0,063	0,051
MyIntrovert	0,048	0,953	0,001	Integr-Iyengar	0,197	0,844	0,001
				Integr-Mind	-0,102	0,919	0,000
				Iyengar-Mind	-0,298	0,767	0,001
MyExtrovert	0,389	0,679	0,008	Integr-Iyengar	-0,869	0,388	0,011
				Integr-Mind	-0,477	0,635	0,003
				Iyengar-Mind	0,409	0,684	0,002
MyInterpret	1,110	0,333	0,022	Integr-Iyengar	-1,333	0,187	0,025
				Integr-Mind	-1,181	0,242	0,020
				Iyengar-Mind	0,222	0,825	0,001
PCL	0,347	0,708	0,007	Integr-Iyengar	-0,822	0,414	0,010
				Integr-Mind	-0,158	0,875	0,000
				Iyengar-Mind	0,616	0,540	0,006
SF36Ment	1,080	0,343	0,021	Integr-Iyengar	1,021	0,311	0,015
				Integr-Mind	1,384	0,171	0,027
				Iyengar-Mind	0,479	0,633	0,003
SF36Phys	0,216	0,806	0,004	Integr-Iyengar	-0,651	0,518	0,006
				Integr-Mind	-0,384	0,703	0,002
				Iyengar-Mind	0,273	0,786	0,001
SR	0,285	0,753	0,006	Integr-Iyengar	0,699	0,487	0,007
				Integr-Mind	0,212	0,833	0,001

	Iyengar-Mind	-0,528	0,599	0,004

Between group comparison (ANOVA)

Diff V0 V2

Questionnaire	Diff V0 V2 ANOVA F	p	eta2	Post-hoc Comparisons comp	t	p	eta2
PSS	1,439	0,242	0,028	Integr-Iyengar	1,062	0,292	0,017
				Integr-Mind	1,653	0,104	0,039
				Iyengar-Mind	0,678	0,500	0,007
ASPConscious	0,276	0,759	0,006	Integr-Iyengar	-0,754	0,454	0,008
				Integr-Mind	-0,499	0,619	0,004
				Iyengar-Mind	0,234	0,816	0,001
ASPReligiousOrient	1,400	0,251	0,028	Integr-Iyengar	-1,486	0,142	0,031
				Integr-Mind	-1,261	0,212	0,022
				Iyengar-Mind	0,341	0,734	0,002
ASPSearchWisdom	0,290	0,749	0,006	Integr-Iyengar	0,620	0,537	0,006
				Integr-Mind	0,705	0,483	0,007
				Iyengar-Mind	0,054	0,957	0,000
ASPTranscendence	0,278	0,758	0,006	Integr-Iyengar	0,568	0,572	0,005
				Integr-Mind	0,703	0,485	0,007
				Iyengar-Mind	0,026	0,979	0,000
BLR	0,467	0,628	0,009	Integr-Iyengar	0,317	0,752	0,001
				Integr-Mind	0,911	0,366	0,012

				Iyengar-Mind	0,608	0,545	0,005
BLR1	0,263	0,769	0,005	Integr-Iyengar	-0,595	0,554	0,005
				Integr-Mind	0,145	0,885	0,000
				Iyengar-Mind	0,663	0,510	0,006
FFA	0,145	0,865	0,003	Integr-Iyengar	-0,540	0,591	0,004
				Integr-Mind	-0,362	0,718	0,002
				Iyengar-Mind	0,158	0,875	0,000
HADSA	0,392	0,677	0,008	Integr-Iyengar	-0,018	0,986	0,000
				Integr-Mind	0,766	0,447	0,008
				Iyengar-Mind	0,737	0,464	0,008
HADSD	0,083	0,920	0,002	Integr-Iyengar	0,440	0,661	0,003
				Integr-Mind	0,140	0,889	0,000
				Iyengar-Mind	-0,258	0,797	0,001
MAIAAufmerkRegula	0,279	0,757	0,006	Integr-Iyengar	0,389	0,699	0,002
				Integr-Mind	-0,390	0,698	0,002
				Iyengar-Mind	-0,706	0,483	0,007
MAIABemerken	0,272	0,762	0,005	Integr-Iyengar	0,587	0,559	0,005
				Integr-Mind	-0,122	0,904	0,000
				Iyengar-Mind	-0,683	0,497	0,007
MAIAEmotGewahr	2,293	0,106	0,044	Integr-Iyengar	-0,458	0,649	0,003
				Integr-Mind	-1,975	0,052	0,053
				Iyengar-Mind	-1,624	0,109	0,038

MAIALeibHören	1,130	0,327	0,022	Integr-Iyengar	1,102	0,274	0,017
				Integr-Mind	-0,378	0,707	0,002
				Iyengar-Mind	-1,475	0,145	0,031
MAIANichtAblenken	1,117	0,331	0,022	Integr-Iyengar	-1,051	0,297	0,016
				Integr-Mind	0,400	0,691	0,002
				Iyengar-Mind	1,400	0,166	0,029
MAIANichtSorgen	0,038	0,963	0,001	Integr-Iyengar	0,182	0,856	0,000
				Integr-Mind	0,266	0,791	0,001
				Iyengar-Mind	0,091	0,928	0,000
MAIASelbstregula	0,352	0,704	0,007	Integr-Iyengar	-0,745	0,459	0,008
				Integr-Mind	-0,733	0,466	0,008
				Iyengar-Mind	-0,077	0,939	0,000
MAIAVertrauen	1,467	0,236	0,029	Integr-Iyengar	-0,522	0,603	0,004
				Integr-Mind	-1,812	0,074	0,045
				Iyengar-Mind	-1,081	0,284	0,017
MBICY	2,690	0,073	0,052	Integr-Iyengar	-1,653	0,103	0,039
				Integr-Mind	0,800	0,427	0,009
				Iyengar-Mind	2,113	0,039	0,062
MBIEX	2,969	0,056	0,057	Integr-Iyengar	1,219	0,227	0,022
				Integr-Mind	2,449	0,017	0,081
				Iyengar-Mind	1,181	0,242	0,020
MBIPE	1,686	0,191	0,033	Integr-Iyengar	-0,180	0,858	0,000

				Integr-Mind	-1,650	0,104	0,038
				Iyengar-Mind	-1,484	0,143	0,032
MyIntrovert	0,049	0,952	0,001	Integr-Iyengar	-0,259	0,797	0,001
				Integr-Mind	-0,264	0,793	0,001
				Iyengar-Mind	-0,029	0,977	0,000
MyExtrovert	0,916	0,403	0,018	Integr-Iyengar	-1,404	0,165	0,028
				Integr-Mind	-0,727	0,470	0,008
				Iyengar-Mind	0,600	0,551	0,005
MyInterpret	0,561	0,572	0,011	Integr-Iyengar	-1,072	0,287	0,016
				Integr-Mind	-0,218	0,828	0,001
				Iyengar-Mind	0,795	0,430	0,009
PCL	0,881	0,418	0,017	Integr-Iyengar	-0,049	0,961	0,000
				Integr-Mind	1,081	0,284	0,017
				Iyengar-Mind	1,187	0,240	0,021
SF36Ment	0,557	0,575	0,011	Integr-Iyengar	1,068	0,290	0,017
				Integr-Mind	0,134	0,894	0,000
				Iyengar-Mind	-0,785	0,435	0,009
SF36Phys	0,442	0,644	0,009	Integr-Iyengar	-0,802	0,425	0,009
				Integr-Mind	0,099	0,922	0,000
				Iyengar-Mind	0,854	0,397	0,011

SR	0,147	0,864	0,003	Integr-Iyengar	0,027	0,979	0,000
				Integr-Mind	0,489	0,627	0,003
				Iyengar-Mind	0,479	0,634	0,003

Plot Practice Time

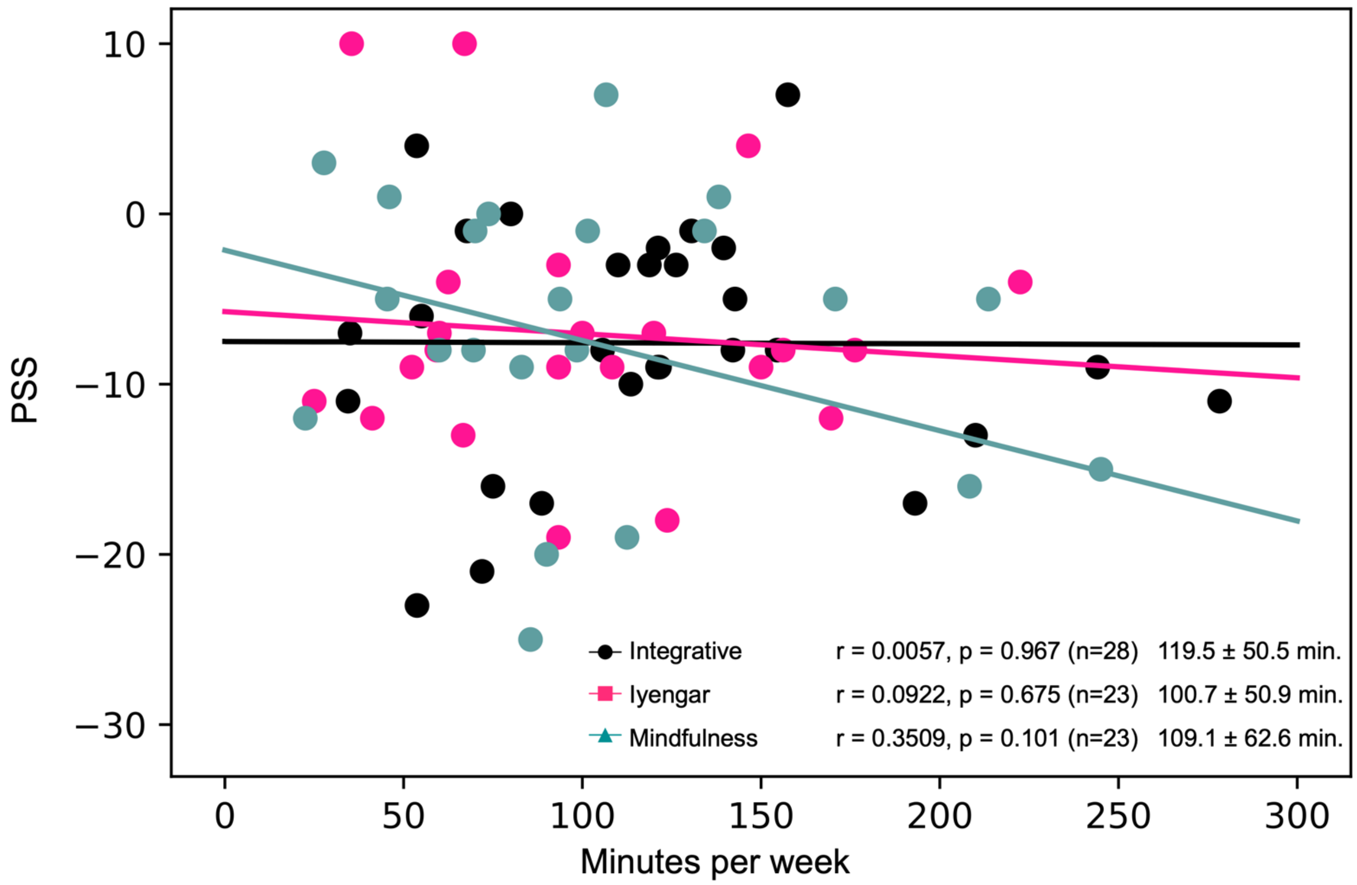

The graph shows the individual magnitude of PSS reduction between dates V0 and V1 as a function of, or in relation to, the time spent practicing (in minutes per week). Given were 30 min. daily, which corresponds to 210 min./week. The data show a slightly greater reduction in stress (PSS) with longer practice time especially for the mindfulness participants, however, this did not reach statistical significance (r = 0.167, p = 0.156, for the n= 76 subjects who entered their data). The effect of exercise time is p = 0.15.

Results – Qualitative Interviews

1 Changes

Using Mayring's content analysis, three main categories with thirteen subcategories were formed.

These are shown in the following diagram:

Changes

- Everyday life
- Self-care
- Psychological
- Somatic
- Change as a process that takes time

Course evaluation

- General evaluation
- Educational share
- Course instructor

Other

- Hopes / Fears
- Lockdown situation
- Previous experience
- Future

1.1 Change everyday life

The extent to which the different courses had an effect on the everyday life of the test persons becomes clear in this category. Many of the participants experienced a change in their everyday life.

General changes in everyday life

The two participants in the integrative course described that the routine that the course gave them got them out of their everyday rut and experienced that it is possible to take time for oneself. Even if the daily routine seems full, *it is possible, "if you want to, it is possible, then you can shovel everything free"* (I4 Integrativ, 54).

> *"I think that was also a great merit of this course, that you are taken out of this rut, out of this everyday life [...] Now it is not only something theoretical, self-care and care and be good to yourself, but you were virtually forced to do that now in the course with these exercises.* (I3 Integrative, 44)

In addition, the participants used to deal with other things:

> *"So the mindfulness too, just no radio, no TV and all the windows closed and this incredible quiet with a candle and just being there and finding one or two* ***exercises to let go.****"* (I5 Iyengar, 46)

Mindfulness in everyday life was also described by another participant, but in a different reference:

> "*Yes, so this mindfulness of what's happening to me and where I am right now and I'm trying to control that better. [...] **And now I always try to be aware of** how each step is happening, what I'm doing and try not to get distracted. That's not always successful, but that's what I try to do.*" (I6 Mindfulness, 36)

From the data of the Iyengar course participants, it is clear that everyday life had not changed as a result of the course so far:

> *"My daily life is, it went on as it was before, or still is now. **But I include an hour for myself**"* (I5 Iyengar, 17)

> *"Nah there was never anything like that. It didn't **affect** my **daily life that much**. It didn't effectively affect the day to day. It was just way too little for that too and the rest of life I think was too stressful."* (I1 Iyengar, 36)

Apart from the Iyengar participants, for whom the course brought more stress into their everyday life, this problem was not mentioned by any other course participants. The long journeys were said to be "exhausting" and they were "a bit overwhelmed" (I5 Iyengar, 6).

> *"that's probably **even more stressful** [...] because you have to drive there, then you're there for an hour and a half, then you have to drive back and then it's totally stressful with the kids, quick to bed and so and you have the homework. That's what I thought. And then it started like that and of course it was stressful because I had to totally rush to go there."* (I1 Iyengar, 4)

Daily practice

As presented earlier, for the participants, daily practice was a big change in their daily lives. The active and consistent practice "has a good effect [,] it feels good" (I4 Integrative, 26) and *"It is **sustainable and has an effect.** "*(I3 Integrative, 40), some of the participants described.
Nearly all reported that it is possible to take time "daily" (I2 Mindfulness, 20) and regularly, and "the fixed date" (I3 Integrative,8) serves as motivation to do it "consistently" (I5 Iyengar, 9; I3 Integrative, 37; I6 Mindfulness 18).

> *"Got a lot of things done, but never an hour really just for me. And I have that now, I've learned that it's nice, does good, and is important."* (I5 Iyengar 23)

> "*The execution and the exercises, that was new and easy, that I learned to approach this actively. And that I also saw that if I do that consistently, that also has an effect.* " (I3 Integrative, 37)

Only one participant, who described Iyengar yoga as "boring" (I1 Iyengar, 37) because it was always the same exercises, commented rather negatively on her practice at home and would not choose this style of yoga herself:

> *"But there, of course, you don't do an hour and a half, but then you kind of do half an hour like that between cooking dinner and correcting homework."* (I1 Iyengar, 12)

1.2 Change self care

Most participants felt a change in self-care, in "paying more attention to themselves" (I6 Mindfulness, 12). They reported taking more time for themselves and paying more attention to their needs as a result of the course. Self-care *"naturally falls by the wayside in everyday life"* (I3 Integrative ,44) *"and now I take it and now I realize how good it is"* (I5 Iyengar, 21), that one can *"actively [do] something about it oneself"* (I3 Integrative, 40).

> *"**I didn't know before that it would be my turn.** And now I know that, now I find that beautiful and need that. And I do this and this is important. [...] I have never done anything for myself before with the consistency that I have done in these hours, so concentrated and so consistent. And also with such dedication and such passion."* (I5 Iyengar 41-44)

Also through the mindfulness course, both interviewed participants were encouraged to do something good for themselves and have since regularly taken a warm bath, which, according to the course instructor, "releases messenger substances [that] contribute to well-being" (I2 Mindfulness, 28):

> *"But a little bit, I have achieved **that I allow myself more**. [...] I have a Jacuzzi in the bathroom, I've never used it, for 15 years, [...] because I didn't allow myself that. [...] because that was a pampering for me and I'm not allowed to do that. I've overcome those little things now and that's what I do now."* (I6 Mindfulness, 11)

Both participants of the integrative yoga course mentioned that they had learned to take care of themselves and that they could pass this on to others and "really just recommend it to everyone" (I4, 28).

> *"So just taking care of myself, and through that just then passing that on to others."* (I3 Integrative, 21)

Dealing with stressors had also changed, according to one mindfulness participant:

> *"From noon on, I now don't read any mails [...] that I think might keep me busy during the night."* (I6 Mindfulness, 23)

1.3 Psychological changes

In mindfulness, mental training and especially calming the mind plays a major role; in this regard, the interviewees discussed the change in the psyche.

General mental changes

The participants reported that they had "become closer to themselves" (I6 Mindfulness 3), described the course as "very helpful, mentally speaking" (I4 Integrative 62, cf. I5 Iyengar 24-25) and had "noticed positive changes quite quickly" (I2 Mindfulness, 4). It was also mentioned with enthusiasm that attention was drawn to topics such as spirituality and bodily awareness, topics with which one "generally doesn't really [deal with] so necessarily" (I4 Integrative,20).
In addition to the participants for whom the yoga or mindfulness practice was described as "my salvation" (I2 Mindfulness, 4), however, some of the practices did not achieve the desired effect. One participant, when asked if the course was helpful for her problems, "this fatigue, this[n] state of exhaustion" (I6 Mindfulness, 27), responded as follows:

> "*Yes so a little bit. Not as much as I would have liked, and as I heard with the other women*." (I6 Mindfulness, 38)

However, the mindfulness course was also able to help the participant

"on the right track" (I2 Mindfulness, 33).

Other participants were also able to perceive the course assigned to them as helpful for the psyche and to fight the feeling of "no way out" anymore, "this black hole" (I3 Integrative, 26), and to escape from stress:

> *"Well, these symptoms I had back then when I was really racing on the hamster wheel [...], it's also such a creeping and imperceptible process that you actually expect more and more of yourself because it's still working* ***without stopping*** *and then you put off a lot of things, like I'm going to yoga, of course there's* ***no time for*** *that* ***anymore. You put that off to*** *sometime."* (I4 Iyengar, 31)

Dealing with stress

The interventions were able to support many participants in dealing with stressors. The participants were able to build a distance to them: They helped to "decatastrophize experiences" (I6 Mindfulness, 39) as well as to learn that one is not "at the mercy" and that there are possibilities to get out of the "black hole" (I3 Integrative, 6). Furthermore, the topic of self-efficacy was an important issue during the stress exposure - it was important for one participant from Integrative Yoga to know that she "can actively do something and that she is not so powerless at the mercy of it all" (I4 Integrative, 25). The other participant in the integrative yoga course was also shown ways to get out of a stressful situation and help her to overcome the "rather difficult time" (I3 Integrativ, 3):

> *"I really had the feeling that I was in over my head and that I couldn't cope with my* ***own strategies*** *for coping with professional stress, and I have to say that* ***yoga helped*** *me a lot, as did the* ***accompanying words*** *[...]. So I actually noticed a clear improvement after just a few days."* (I3, Integrative 3)

> *"Tried to distance myself, when I felt that now complicated situations arise I withdrew and did not react immediately, but let it affect me and then I reacted accordingly, but more calmly and not as emotionally as until now."* (I6 Mindfulness, 3)

The integrative course was also able to help in dealing with professional stress. In the stressful phase of life before the Integrative Course, professional stress caused tension; now the participant can approach such problems again with calmness (cf. I3 Integrative, 57). In this respect, the mindfulness course was perceived as helpful and helped in dealing with occupational stress in a different way:

> *"I don't want to do this to myself anymore because it doesn't get me anywhere and I also want [...] to pursue myself and my interests. Yes,* ***I've always dutifully done everything and*** *accepted* ***everything*** *and now I thought, no!"* (I6 Mindfulness 29-31)

> *"In the current situation, in the job I do. [...] It's critical, I don't know exactly how things will go in January. [...] And I'm* ***totally relaxed about*** *it.* ***Something will happen.*** *And don't break down now [...] I had also formulated it that way,* ***to be*** *in the* ***here-and-now.****"* (I2 Mindfulness, 56-59)

The participant and the participant of the mindfulness course express themselves as only to what has changed in the conscious encounter of thoughts. It is possible that negative thoughts, respectively "these stressors" are replaced with positive "mindfulness thoughts [...]" (I2 Mindfulness, 17). Participants in the mindfulness course perceived this aspect of the course very differently, as shown by the following quote:

> *"Then I can't change that feeling. So I'm very controlled, controlled by the head and* ***I know that doesn't change*** *and then I have no influence over that mechanism."* (I6 Mindfulness, 9)

While this participant could be motivated to "*try[n] to distance* herself *and change [her] perspective."* (I6 Mindfulness, 25) as well as becoming closer to herself, however, she is "*very controlled and knows that doesn't change and then I have no influence over that mechanism"* (I6 Mindfulness, 9).
One Iyengar course participant did not comment on any changes in stress experience, however, mentioned several times that the course was too *"strict and severe*" for her (I1 Iyengar, 5). However, the other participant in this course was able to experience that there are ways to *"deal with anxiety or stress"* through practicing yoga daily (I5 Iyengar, 41). However, this one also reported the following:

> *"But when you are so burdened with anxiety, yoga doesn't help relieve the stress from it either."* (I5 Iyengar, 33)

Serenity, More Relaxed, More Mindful

Greater serenity and relaxation in everyday life were perceived by almost every participant. In particular, the participants reported a more relaxed attitude toward the future, which was perceived as frightening by some participants before the courses. However, a more relaxed approach to the family, professional environment and fellow human beings in general was also reported.
Integrative yoga could also lead to the participants being able to "be more composed" (I3 Integrative, 23) in certain situations:

> *"So it certainly also plays a role in the fact that I am much* ***more attentive in the*** *end, that I can respond to others differently. In dealing with others, it definitely helps me to be more relaxed, which was also my goal, to be* ***more relaxed.***
>
> *Get that* ***relaxed serenity****."* (I4 Integrative,49)

In the Iyengar class, the experiences diverged somewhat. One of the two participants noticed that the calm she takes in the meantime,
"**serene** [is] and how **relaxed** too." (I5 Iyengar, 21)

The second student in the Iyengar class also reports relaxation, but emphasizes that it lasted only for the moment:

> *"In the end I was really totally* ***relaxed*** *after that hour and a half and was totally* ***relaxed, for the moment,*** *because then I had to get on the bike then I had to race home. Then I had to quickly, quickly [put] the kids to [bed]."* (I1 Iyengar, 8)

It should also be added that she is "more of a fidgety person" (I1 Iyengar, 4) who relaxes when she moves.

"And in the hours where we were moving, I found it more relaxing than those quiet hours." (I1 Iyengar, 17)

The more conscious perception of, for example, objects that were no longer found in the past (cf. I6 Mindfulness, 36) or dangers that are now faced more consciously was achieved by practicing mindfulness:

"Yes, so these are things, when I look back at my life, at the past, that there were also critical moments where I think I intuitively avoided dangers, where I now sometimes have the impression, based on these insights that I have gained, that I do this ***more consciously in*** *part"* (I2 Mindfulness, 55)

One participant in the Iyengar course described the online courses, which could no longer take place in the studio due to Corona, as not relaxing:

"So that was no longer relaxing for me at all. Because the children were somewhere and it took an hour and a half. ***At home I'm not relaxed*** *when I think ah now you should do this, and now you still have to do the dishwasher."* (I1 Iyengar, 18)

One participant of the integrative course perceived the changes as subtle. Precisely because a lot had changed in her case, many things had happened at the same time, there was a change in her. That this had a *"very relaxing effect on* her" (I4, 14),
"It's a mixture of the whole thing" (I4, 25) and, in their opinion, can have various causes:

"So it's just good for me to get a bit of serenity, more than it was in the past, but as I said, you have to document it over a longer period of time in order to draw comparisons and perceive the differences directly, because it's just so imperceptibly small-scale." (I4 Integrative, 51)

Likewise, the participant of the mindfulness course mentioned this complexity of the mode of action:

"So it's already clear to me that everything is very complex. But I think it's amazing and I think it's great and I'm really totally happy with it." (I2 Mindfulness, 31)

Dealing with fear

The majority of respondents expressed that their management of their own fears had improved. One participant in the mindfulness course, in a situation where she was worried about what others might think of her, was able to "stay totally loose" (I6 Mindfulness, 33). The other participant in the same course was also able to "free himself from his fear cage [...]" (I2 Mindfulness, 51) and "be relatively free of anxiety over a very long period of time" (I2 Mindfulness, 23).
Practicing Iyengar yoga daily was able to show another participant ways to deal with her "anxiety or stress" (I5 Iyengar, 41).
Even situations such as the current pandemic could be approached more calmly and were not characterized by fear:

"I mean we came now with the course of all things into this whole Corona time, which one uu beginning, I do not know, how it went to them however, already made so a little

*bit afraid. I think that I somehow dealt with it a bit **more calmly** than I would have otherwise." (I3 Integrative, 23)*

Emotional solution

Mainly the mindfulness course caused a very deep emotional confrontation with oneself. Thus, meditating and practicing relaxation exercises could help both with the grief processing of the death of a
family member, as well as to question and partially break through learned structures of upbringing:

> *"From my upbringing, there was only one's own performance and not one's own well-being, but that would be something negative to feel good about, and that's how I was brought up, have been working on distancing myself from that for a long time, and in these 12 weeks I've been focusing more on that"* (I6 mindfulness, 11)

The other mindfulness practitioner was able to feel a "fundamental shift" with the help of the course exercises:

> *"And of course it wasn't always just joyful, there were also moments where I **went very deep inside myself**, where it was painful, where there were tears. But then there were also moments where I really noticed a fundamental change. And where I really became aware that I had done too much to myself before. And one of the **greatest moments** was that after a meditation I stood there on my mat and started to cry and asked aloud to my body for forgiveness for what I had done to it for 20 years in the job in which I now work. Nobody had told me to do that, that came from within, and I was very aware of that at that moment, that that came from me. And there were a few things like that then, during the course and even later."* (I2 Mindfulness 12)

1.4 Health changes

The differences in perceived physical changes are large. Participants report relief from health problems, a "completely different attitude" (I2 Mindfulness, 62) but also no physical changes through the Study interventions.
For example, no improvements were seen in osteoporosis (cf. I5 Iyengar, 20, cf. I6 Mindfulness, 27).

Very differentiated experiences on the physical changes can be drawn from the data of the mindfulness group. On the one hand, a stress symptom mentioned, a trembling in the hand occurring during stress, could not be eliminated (cf. I6 Mindfulness, 15-16). On the other hand, in the case of psoriasis, an autoimmune disease, the "relaxation" achieved (I2 Mindfulness, 28) made the "*external symptoms [...] go away to almost zero within 14 days."* (I2 Mindfulness, 27) Respondents emphasized physical improvements "diffuse[d] gastrointestinal problems" (I3 Integrative, 25) and back pain as well as a generally better physical feeling:

> *"I had increased **migraines**, so kind of every two weeks, that's gone now, so maybe once in a quarter still like it was before."* (I3 Integrative 25)

*"So on the one hand **health-wise** I noticed that through the exercises and also through the relaxation exercises and also through the Bodyscan but also through my modified morning exercises. [...] over a very long period of time, for example, to keep back **pain** very small. Or if they occur, then I have exercises so that I no longer have them the next day. Also with **tennis elbow**, I had found effective exercises how I can treat that very well for myself"* (I2 Mindfulness, 25).

The change in flexibility could be experienced by a participant of Integrative Yoga as well as a positive change in the "shoulder-neck area" (I4 Integrative, 43) .
Only one participant spoke of a weight loss. He reported losing five kilograms, not because of a change in eating habits, but because of *"mental confrontation"* (I2 Mindfulness, 64).

Sleep Change

While participants in the stress reduction programs did not notice any difference in sleep quality (cf. I4 Integrative 67, I5 Iyengar 51), others who previously suffered from sleep disorders reported an improvement:

*"It began that the **quality of sleep gradually** got a little better."* (I3 Integrative, 6)

One participant in the mindfulness course, however, complains of sleep problems, which are very stressful for her, despite her current lack of stress (cf. I6 Mindfulness, 16).

Process that takes time

According to the interviewees, one has to deal with physical or mental
Being patient with changes. Two participants emphasize that it is a process and that "*maybe [...] it is just too short"* (I5 Iyengar, 60) to be freed from one's complaints.

"A process [...] that simply also takes time, that doesn't happen from one moment to the next" (I4 Integrativ, 49).

2 Course evaluation

2.1 General evaluation

In general, the different courses were perceived as very positive and helpful.

However, one participant did not benefit from the course. "*It didn't affect [her] daily life that much"* (I1 Iyengar, 37). She perceived the very strict and rigorous yoga, as she described it, as *"interrupted*" (I1, 6). The yoga direction she came from was *"much more playful, tantric, in the sense of, celebrating the joy of life' [...] that was not so strict*" (I1 Iyengar, 26). Even though she described it as "*interesting"* (I1, 28), it was "*too tight, not playful enough"* (I1, 28) and she had been *"totally disturbed*" by some of the "*tools in Iyengar yoga"* (I1 Iyengar, 27).

However, Iyengar yoga was not only described as "boring" (I1, 7), but...

"then also had its nice" (I1Iyengar 35). This daily "challenge" and the "joy of having accomplished [the exercises]" (I5 Iyengar, 74) motivated another participant of the Iyengar course to do something good for herself:

"I really had to go there, I was drawn to it and I wanted to know, I wanted to find out for myself: does it bring you what you don't even know yet that it can or should bring you." (I5 Iyengar, 44)

In Integrative Yoga, the effectiveness of simplicity was addressed and,

"that it is not boring at all" (I4 Integrative, 55). The two participants agreed that this style of yoga had done them good:

"And it has a good effect and it feels good." (I4,25)

"But there was actually no moment, so there was no second where I thought, this is not good for me now. Or that it would cause me pain in the movement, no, there was no such thing" (I3 Integrative, 13).

The exchange, which was not mentioned by the Iyengar course participants, is described by the participants of the Integrative course as *"enriching and supportive"* (I4, 5-6), as it gives the feeling *"you are not so all alone in the world, it is not an exceptional problem, but quite a few have it."* (I3, 49)

The mindfulness course also "fell on such fertile ground" (I2 Mindfulness, 2) that "I [...] cannot say anything negative about the course now" (I2 Mindfulness, 40) and the course was perceived "as **enrichment**" (I6 Mindfulness, 41).

2.2 Educational part

The Iyengar course, according to one participant, could not offer any assistance for stressful situations, for that she would have liked to get "tips and tricks" (I1 Iyengar, 54). She would have expected "suggestions [...] for relaxation, a bit of theory for relaxation" (I1 Iyengar, 51), since what was learned was "not directly applicable, so to speak" (I1 Iyengar, 23).
A participant of Integrative Yoga justifies the sustainable, positive effect with these

"accompanying explanations. The [...] psychological in quotation marks" (I3 Integrative, 42).

*"But I think it was just also **the accompanying, also the tips**. That which he also gave us along the way, certain Taoist wisdom."* (I3 Integrative,11)

2.3 Course instructor

From each course, the instructor was praised. They were "enthusiastic" (I5 Iyengar, 3). It was said that *"I thought the instructor did a pretty good job"* (I 2 Mindfulness, 39) and that one felt "professionally [...] accompanied" (I6, 39). Another participant says that the course instructor always gave her new courage and that she "felt at home" (I3, 51).

"He was believed to live what he said because he also picked up every one of us." (I3 Integrative, 52)

2.4 Negative aspects of the courses

One participant felt that the Iyengar course was "too little" (I1 Iyengar, 47). She would have liked more exercises to practice at home and "did not have the feeling that the whole body [...] moved through, but only so partially" (I1 Iyengar, 47).

"That was also a **problem of** the course. It was kind of like 8 or 10 exercises and we did these 12 hours of just these 10 exercises. I found that a little bit little. I would have liked to have had just **more variations.** Something else, another exercise." (I1, Iyengar 43)

The other participant of the Iyengar course also emphasized that it was "too little" for her:

"And it was too little. I got a lot out of it, but it was out of the 12 times that it should have been, suddenly it was only 6 or 7, that was a little bit of a mess." (I5 Iyengar, 12)

One participant in integrative yoga also reported that it was "always the same simple exercises," but that she did not find them boring but "pleasant" (I4 Integrative, 55).
Furthermore, it was thematized that it is difficult to respond to "such different people" (I1 Iyengar 71) and to consider the different levels. In the mindfulness course, one participant also had the feeling of being *"one step [...] further ahead"* (I2 Mindfulness, 10).
One participant in the mindfulness course expressed her disappointment in herself. Even though she *"participated with the best intentions [and] consciously* did not *block anything"* (I6 Mindfulness, 18), "*the other women [...]* were *enthusiastic and could feel a lot. [But] that was not the case with [her]"* (I6 Mindfulness, 5).

"So we started with this **body scan**, I could feel very little, almost nothing. [...] I could not concentrate. So I was away with my thoughts, I was distracted, and then I always controlled myself to stay with it, but that didn't really work" (I6 Mindfulness, 4).

Other participants, however, experienced this quite differently. Especially the Bodyscan was emphasized by many as positive:

"Well, for example, I found this body scan very good and then the relaxation I just find good. And then just being with yourself, just breathing these simple things under guidance of course, that's just super pleasant." (I4 Integrative, 46)

3 Other

3.1 Hopes/Fears

For example, the Iyengar course could not fulfill the hope of *"compensating a little bit with yoga"* (I5 Iyengar, 20) as well as that there was no change in dealing with stressful situations (I5 Iyengar,17). Nevertheless, yoga was able to provide great serenity and relaxation, as well as daily motivation, fascination, and challenge (cf. I5, 81).

"That's such a positive thing that I took away. Keep going. You can just keep going, keep at it. It certainly hasn't done any harm. And I do everything that is good for my health, that gives me a little bit of serenity in my daily life." (I5 Iyengar, 81)

The other participant missed in Iyengar Yoga that stress was not addressed (cf. I1 Iyengar, 22). Thus, it could not help her in terms of stress, but she could experience a certain "*relaxation, for the moment*" (I1 Iyengar, 7). *"But if it's super stressful, then a yoga class like that can't pull anything out."* (I1 Iyengar, 39)
In the mindfulness group, the course was able to bring about very positive change in one participant in particular. He *"got involved* without expectations, *without thinking much about it*" (I2 Mindfulness, 7) and was thus able to release fears (cf. I2 Mindfulness, 51) as well as to cope better with stress (cf. I2 Mindfulness, 17). He feels a "*great enthusiasm and gratitude*" (I2 Mindfulness, 66) and sees it as a *"lucky circumstance*" (I2 Mindfulness, 3) to have had the opportunity to be a part of

the course. The interview with the participant of the mindfulness course also revealed that the participant had a certain

"Willingness to learn more in this area and to do something for myself" (I2 Mindfulness, 7). On the other hand, the desire to distance oneself from *"negative experiences*" and to evaluate them differently could achieve an effect, but stress symptoms could not be particularly improved (cf. I2, 15-16) and in comparison to the *"other participants I think I have not achieved as much"*(I6, 3).

The integrative yoga course had the *"goal [...] to* achieve *more serenity"* (I4, 49) and was able to free herself from the *"hamster wheel*" (I 4, 25). She emphasizes that *"under this guidance [...] and this binding to implement* it*"* (I4 Integrativ, 28) had helped her a lot. However, she emphasizes always to the fact that many factors have achieved this effect (cf. I4, 25) and that she "already has such a sensitivity" (I4 Integrativ, 44).

The other participant of this course was also able to improve stress symptoms that the subject was suffering from and also meet the expectation:

> *"My expectations were actually fulfilled, so the expectations were actually that I actually get a handling, get tips, get exercises, what I can do myself, how I can become active myself in similar situations and also in the longer term."* (I3, 54)

Before the integrative course, one participant was afraid that it was "too strict, too detached, that you don't arrive in reality" (I3 Integrativ, 54). However, according to her statements, this was quite different in this course and "exactly the opposite" (I3 Integrativ, 56) had happened. In addition, the fear was expressed that she would not be able to take the course for professional reasons. In the end, she was "amazed that it was possible after all" (I4 Integrativ, 22) and "maybe it was only possible because Corona came" (I4 Integrativ 21).

3.2 Lockdown situation

For many participants, the Corona situation was also discussed and that this had an influence on the course. The courses were partly conducted online and had a negative effect on the participants of the Iyengar course, for example, because "being at home [...] is not relaxed" (I1 Iyengar, 19). (I1 Iyengar, 19) and as appointments also had to be cancelled "because the technology did not work" (I5 Iyengar, 12). However, it was emphasized that this was "due to the situation" (I5 Iyengar, 58).

It was also brought up in the Integrative course and in the Mindfulness course that it did not work well with the video lessons and that this "was a bit of a shame [was]" (I3 Integrative, 45; I2 Mindfulness, 39). However, the other participant in the integrative course had found it amazing how well it had worked anyway (cf. I4 Integrative, 6).

3.3 Experience to date

All participants had already had experience with yoga or mindfulness.

Both the participant and the female participant in the mindfulness course mentioned having practiced yoga before starting the course (cf. I6 Mindfulness, 45) and having incorporated *"relaxation postures*" (I2 Mindfulness, 9) into their regular early morning exercises.

Years of practicing Tai Chi and dealing with Buddhist themes as well as personality development even before the Integrative Yoga course were reported (cf. I3 Integrative, 56; I4 Integrative 48).

Previous experience differed in the Iyengar course, one participant had *had "a little yoga experience, [but] not very much*" (I5 Iyengar, 3). The other participant in the comparison had "done a lot of yoga before" (I1 Iyengar, 5).

3.4 Future

What the participants took away for the future overlapped for all participants.

They take away from the courses that they "regularly practice yoga" (I3 Integrativ, 63) or relaxation exercises. That they would like to "consciously continue [to do so]" (I6 Mindfulness, 50) and also want to "*do a course again"* (I4 Integrative, 50). One participant "*hopes that [it] will accompany [him] throughout [his] life*" (I2 Mindfulness, 52) and other participants "*don[t] give up so quickly" (I5 Iyengar,* 71) because they "*hope[n] that it will improve*" (I6 Mindfulness, 20)
Likewise, it is emphasized that they would do it again and also "again in this intensity" (I5 Iyengar, 49)

> *"And would make myself available again at any time."* (I3 Integrative, 65)

The mindfulness course, could not be as helpful with one participant "as [she] would have liked" (I6 Mindfulness, 38) and would try a mix of mindfulness and yoga in the future:

> *"Yes I would try now,* ***this mixture of mindfulness and yoga****. Maybe that would have another dimension. [...] but there with all the exercises, I think you come better to yourself and then it's more open to the meditation and that I could then better receive what is transmitted to me."* (I6 Mindfulness, 45-48)
